# CG200745, an HDAC inhibitor, induces anti-tumour effects in cholangiocarcinoma cell lines via miRNAs targeting the Hippo pathway

**DOI:** 10.1038/s41598-017-11094-3

**Published:** 2017-09-07

**Authors:** Dawoon E. Jung, Soo Been Park, Kahee Kim, Chanyang Kim, Si Young Song

**Affiliations:** 1 Institute of Gastroenterology, Yonsei University College of Medicine, Seoul, Korea; 2 Division of Gastroenterology, Department of Internal Medicine, Yonsei University College of Medicine, Seoul, Korea; 3 Brain Korea 21 PLUS Project for Medical Science, Yonsei University College of Medicine, Seoul, Korea

## Abstract

Cholangiocarcinoma is a devastating malignancy with fatal complications that exhibits low response and resistance to chemotherapy. Here, we evaluated the anticancer effects of CG200745, a novel histone deacetylase inhibitor, either alone or in combination with standard chemotherapy drugs in cholangiocarcinoma cells. CG200745 dose-dependently reduced the viability of cholangiocarcinoma cells *in vitro* and decreased tumour volume and weight in a xenograft model. Administering CG200745 along with other chemotherapeutic agents including gemcitabine, 5-fluorouracil (5-FU), cisplatin, oxaliplatin, or gemcitabine plus cisplatin further decreased cholangiocarcinoma cell viability, with a combination index < 1 that indicated synergistic action. CG200745 also enhanced the sensitivity of gemcitabine-resistant cells to gemcitabine and 5-FU, thereby decreasing cell viability and inducing apoptosis. This was accompanied by downregulation of YAP, TEAD4, TGF-β2, SMAD3, NOTCH3, HES5, Axl, and Gas6 and upregulation of the miRNAs miR-22-3p, miR-22-5p, miR-194-5p, miR-194-3p, miR-194-5p, miR-210-3p, and miR-509-3p. The Ingenuity Pathway Analysis revealed that CG200745 mainly targets the Hippo signalling pathway by inducing miR-509-3p expression. Thus, CG200745 inhibits cholangiocarcinoma growth *in vitro* and *in vivo*, and acts synergistically when administered in combination with standard chemotherapeutic agents, enabling dose reduction. CG200745 is therefore expected to improve the outcome of cholangiocarcinoma patients who exhibit resistance to conventional therapies.

## Introduction

Cholangiocarcinoma is the second most common primary hepatobiliary cancer and has a poor prognosis, with 5-year survival rates in the range of 5% to 15%^1–3^. More than two-thirds of cholangiocarcinoma patients have unresectable disease, and recurrence after curative resection is common. At present, gemcitabine-based regimens are considered as standard treatment for cholangiocarcinoma patients^4, 5^. Despite these advances in treatment, the number of cholangiocarcinoma-related deaths and chemotherapy-refractory cholangiocarcinoma cases continue to increase. Drug resistance in cholangiocarcinoma diminishes the therapeutic effects of gemcitabine-cisplatin; therefore, new agents that increase tumour sensitivity and can overcome drug resistance are urgently needed^3, 6^.

Histone acetylation/deacetylation is an epigenetic mechanism that modulates gene transcription via histone acetyltransferases and histone deacetylases (HDACs)^7^. Hyper- or hypoacetylation of oncogenes and tumour suppressor genes, respectively, is frequently observed in cancer cells^8, 9^. HDAC inhibitors are anti-cancer drugs that acetylate lysine residues in the N-terminal tails of histones, which inhibits their association with DNA and induces the expression of tumour suppressor genes. To date, two HDAC inhibitors—vorinostat and romidepsin—have been approved by the Food and Drug Administration for the treatment of cutaneous T-cell lymphoma^10^. HDAC inhibitors have been shown to suppress cell proliferation *in vitro* and *in vivo* ^11–13^, and can also rapidly alter micro (mi)RNA levels to induce cell apoptosis in breast and pancreatic cancer and cholangiocarcinoma cells^14–16^. MiRNAs are small non-coding RNAs 21 to 23 nucleotides long that engage in post-transcriptional regulation of gene expression by silencing target mRNAs. MiRNA regulate various cellular processes including cell proliferation, differentiation, and apoptosis and their expression is often dysregulated in cancer^17^.

The HDAC inhibitor (E)-N(1)-(3-(dimethylamino)propyl)-N(8)-hydroxy-2-((naphthalene-1-loxy) methyl)oct-2-enediamide (CG200745) was recently developed by CrystalGenomics. HDAC inhibitors are divided into four classes based on chemical structure—i.e., short-chain fatty acids, hydroxamic acids, depsipeptides, and benzamides. CG200745 is an intravenous hydroxamate-based pan-HDAC inhibitor similar to vorinostat^10^ whose anti-proliferative effect has been demonstrated in several types of cancer cells, including prostate cancer, renal cell carcinoma, and colon cancer, either alone or in combination with other chemotherapy drugs. CG200745 was five times more effective than vorinostat in acetylating histone H3 in a colon cancer cell line, and induced acetylation of the tumour suppressor p53, leading to cancer cell death^18^. A previous study showed that treatment with HDAC inhibitor combined with other chemotherapy drugs resulted in an enhanced anti-proliferative effect and reduced toxicity in cholangiocarcinoma cells, and we recently demonstrated that CG200745 has anti-proliferative and synergistic effects in pancreatic cancer cells^19, 20^.

In this study, we investigated the anti-tumour effects of CG200745 in cholangiocarcinoma both *in vitro* and *in vivo* when administered alone or in combination with gemcitabine, 5-fluorouracil (5-FU), cisplatin, and oxaliplatin. We assessed whether CG200745 can overcome the resistance of cholangiocarcinoma cells to gemcitabine and 5-FU, two standard chemotherapy drugs. We also analysed gene expression with cDNA and miRNA microarrays to clarify the molecular mechanisms underlying CG200745 action.

## Results

### CG200745 suppresses cholangiocarcinoma cell proliferation *in vitro*

**Figure 1:**
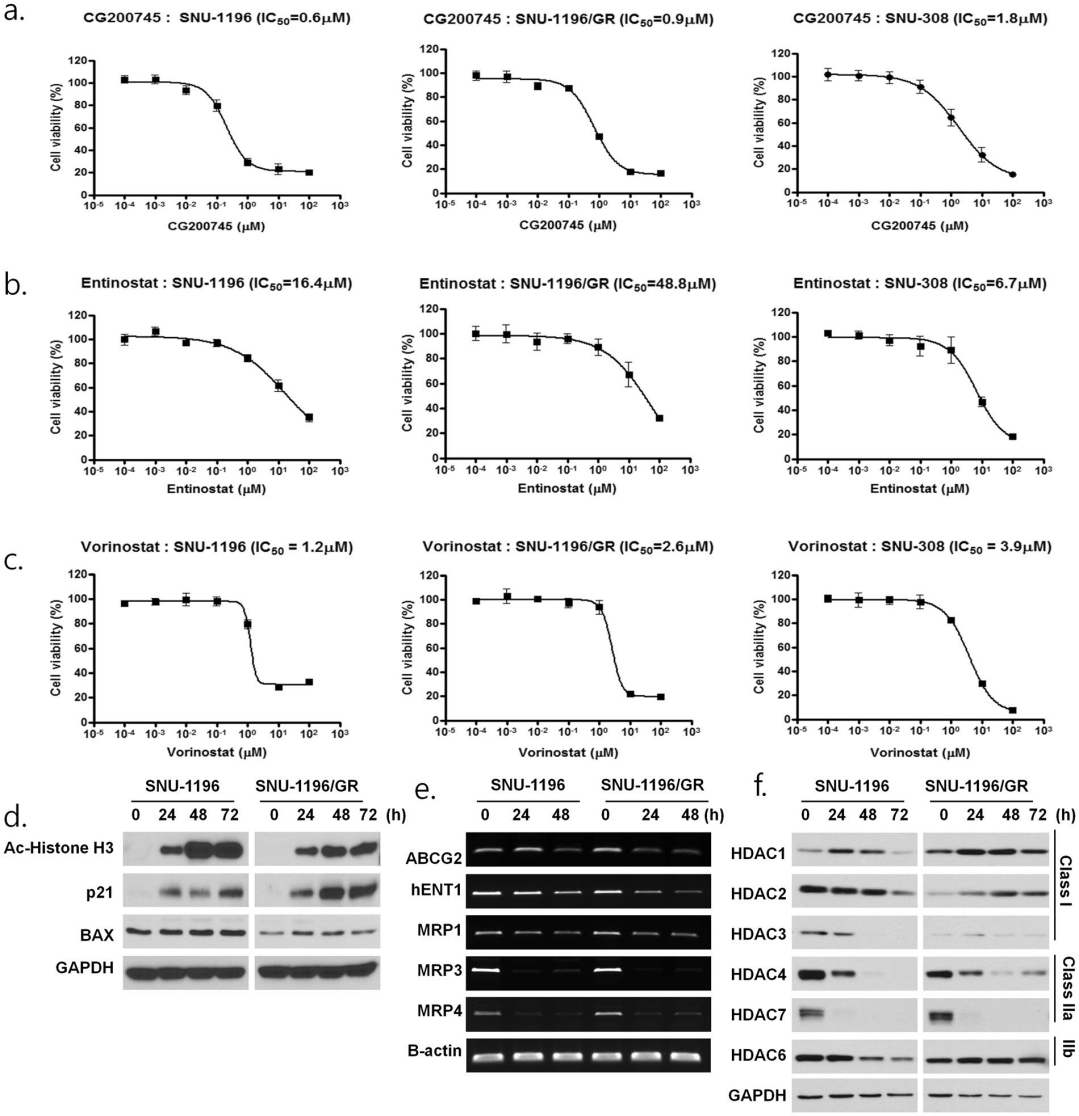
Anti-proliferative activities of HDAC inhibitors against SNU-1196, SNU-1196/GR, and SNU-308 cells. Cell viability was assessed with the MTT assay. (**a**) IC_50_ values of CG200745 were 0.6, 0.9, and 1.8 μM, respectively; (**b**) IC_50_ values of entinostat were 16.4, 48.8, and 6.7 μM, respectively; and (**c**) IC_50_ values of vorinostat were 1.2, 2.6, and 3.9 μM, respectively. In SNU-1196 and SNU-1196/GR cells, (**d**) CG200745 (IC_50_) induced the expression of apoptosis-related proteins p21 and BAX, as determined by western blotting; (**e**) CG200745 (IC_50_) inhibited the expression of multidrug resistance genes, as determined by PCR; and (**f**) CG200745 (IC_50_) altered the expression level of HDAC isozyme, as determined by western blotting.

The sensitivity of SNU-1196, gemcitabine-resistant (GR) SNU-1196 (SNU-1196/GR), and SNU-308 cells to CG200745, entinostat, and vorinostat was determined based on half-maximal inhibitory concentration (IC_50_) values. To assess the effect of CG200745 on drug-sensitive and -resistant cells, we established the SNU-1196/GR cell line with a gemcitabine IC_50_ of 2.291 μM, which is approximately 60-fold higher than that in SNU-1196 (IC_50_ = 0.038 μM) (Supplementary Fig. 1a,b). These cells showed elevated expression of ATP-binding cassette (ABC) transporter ABCG2, as well as proto-oncogenes including c-MET and AKT, the epithelial-to-mesenchymal transition marker N-cadherin, and the tumour-initiating cell surface marker aldehyde dehydrogenase (ALDH) both *in vitro* and *in vivo* (Supplementary Figs 1c,d and 2). CG200745 treatment inhibited proliferation of SNU-1196, SNU-1196/GR, and SNU-308 cells, with IC_50_ values of 0.63, 0.93, and 1.80 μM, respectively (Fig. 1a). CG200745 showed a lower IC_50_ relative to other HDAC inhibitors: vorinostat suppressed proliferation of SNU-1196, SNU-1196/GR, and SNU-308 cells with IC_50_ values of 1.2, 2.6, and 3.9 μM, respectively, whereas entinostat had IC_50_ values of 16.4, 48.8, and 6.7 μM, respectively (Fig. 1b,c). Histone H3 acetylation was increased and expression of the apoptotic proteins p21 and B cell lymphoma (Bcl)-associated X protein (BAX) was induced by CG200745 treatment (Fig. 1d and Supplementary Fig. 3c). Moreover, CG200745 inhibited the expression of multidrug resistance (MDR) genes including ABCG2 and multidrug resistance-associated *protein* (MRP)4; human equilibrative nucleoside transporter (hENT)1; the ABC transporters MRP1 and MRP3; and HDAC class II isozymes including HDAC4 and HDAC7 in SNU-1196, SNU-1196/GR, and SNU-308 cells (Figs 1e,f and. 3d,e).

### Gemcitabine, cisplatin, 5-FU, and oxaliplatin in combination with CG200745 suppress proliferation of cholangiocarcinoma cells

**Figure 2:**
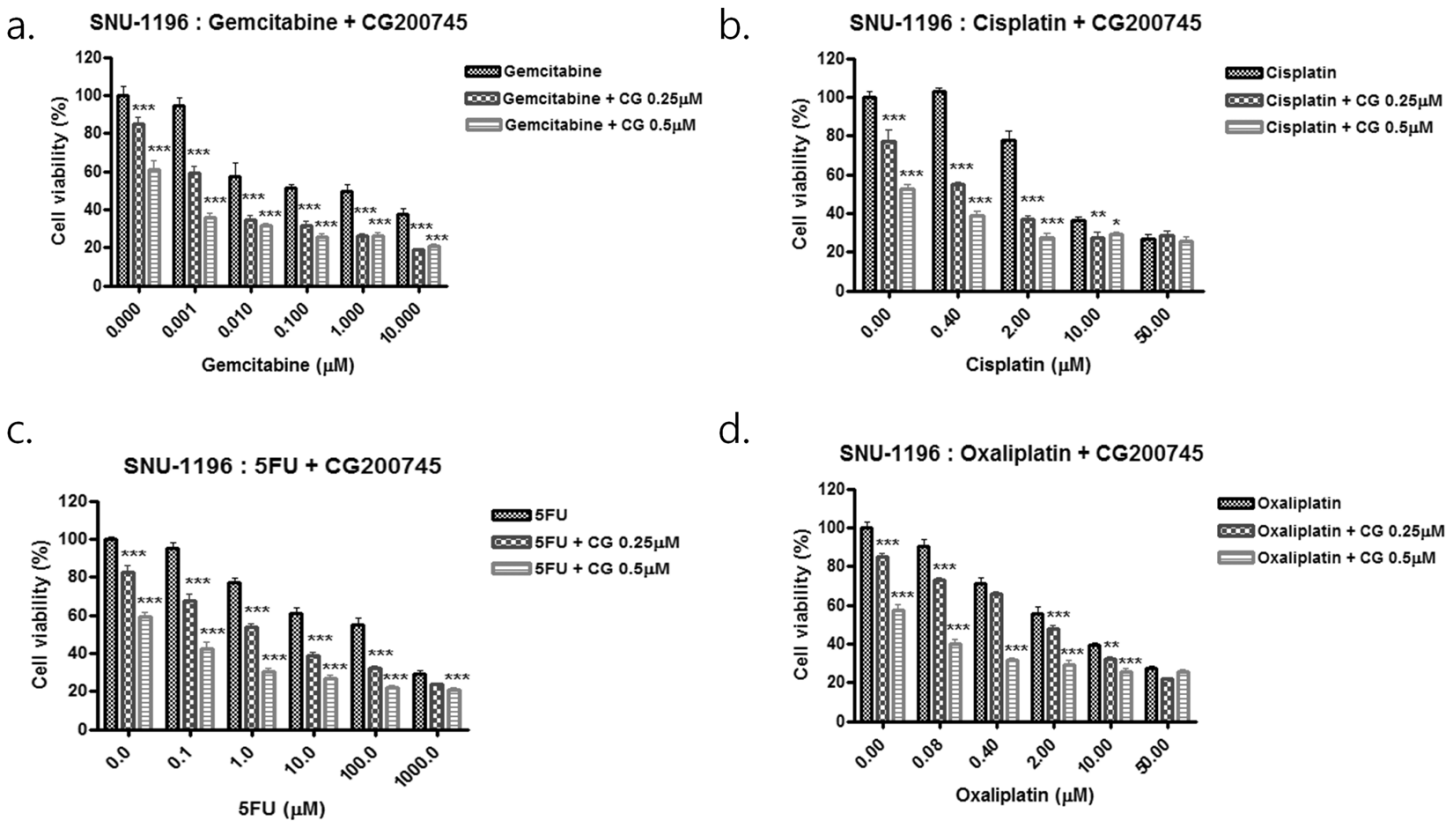
Anti-proliferative effects of gemcitabine, cisplatin, 5-FU, and oxaliplatin in combination with CG200745. SNU-1196 cells were treated with (**a**) gemcitabine, (**b**) cisplatin, (**c**) 5-FU, and (**d**) oxaliplatin along with CG200745 and cell viability was evaluated with the MTT assay. *P ≤ 0.05, **P < 0.01, and ***P < 0.001.

We investigated the combined effects of standard chemotherapy drugs and CG200745 on SNU-1196 and SNU-308 cell proliferation. Each of the drugs tested—including gemcitabine, cisplatin, 5-FU, and oxaliplatin in combination with CG200745 (0.25 or 0.5 μM for SNU-1196 and 0.5 or 1.0 μM for SNU-308)—showed concentration-dependent cytotoxicity in both cell lines (Fig. 2a–d and Supplementary Fig. 4a–d). Upon treatment with 0.25 and 0.5 μM CG200745, the IC_50_ values of gemcitabine (0.038 μM) against SNU-1196 cells decreased to 0.002 and < 0.0001 μM, respectively; those of cisplatin (4.898 μM) decreased to 0.562 and 0.105 μM, respectively; and those of 5-FU (104.713 μM) decreased to 1.862 and 0.01 μM, respectively. Similarly, the IC_50_ values of oxaliplatin (3.236 μM) decreased to 1.585 and 0.25 μM in the presence of 0.25 and 0.5 μM CG200745, respectively (Supplementary Table 1a). In SNU-308 cells, upon treatment with 0.5 and 1.0 μM CG200745, the IC_50_ values of gemcitabine (1.413 μM) decreased to 0.32 and 0.13 μM, respectively; those of cisplatin (3.80 μM) decreased to 2.34 and 1.82 μM, respectively; and those of 5-FU (74.131 μM) decreased to 44.67 and 17.78 μM, respectively. Similarly, the IC_50_ values of oxaliplatin (6.918 μM) decreased to 4.57 and 2.82 μM in the presence of 0.5 and 1.0 μM CG200745, respectively (Supplementary Table 2a).

**Figure 3:**
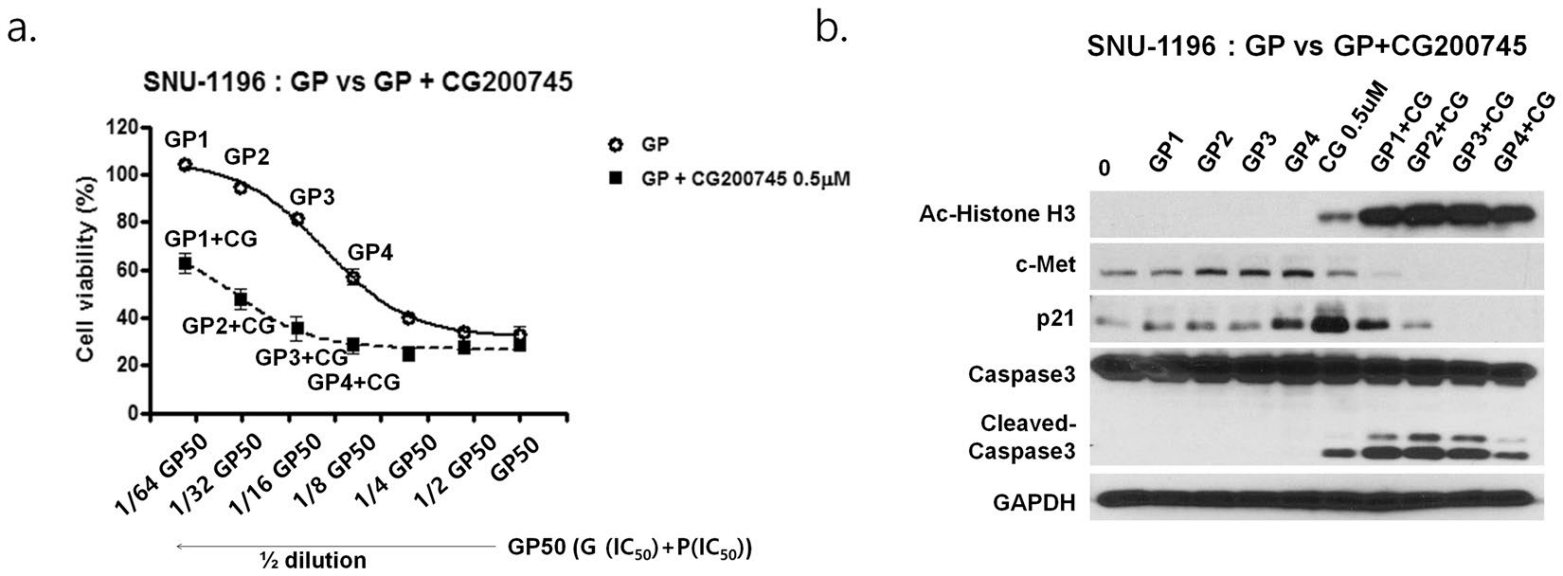
Anti-proliferative effects of gemcitabine plus cisplatin in combination with CG200745. (**a**) SNU-1196 cells were treated with indicated concentrations of gemcitabine plus cisplatin along with CG200745 and cell viability was assessed. G, gemcitabine; P, cisplatin; GP, gemcitabine plus cisplatin; CG, CG200745. (**b**) Cells treated with serial dose of gemcitabine plus cisplatin and CG200745 for 3 days showed downregulation of c-MET and upregulation of cleaved Caspase-3. GP50, gemcitabine IC_50_ plus cisplatin IC_50_; GP1, 1/64 dilution of GP50; GP2, 1/32 dilution of GP50; GP3, 1/16 dilution of GP50; GP4, 1/8 dilution of GP50.

We used CompuSyn software to calculate combination index (CI) and dose reduction index (DRI) to analyse the additive and synergistic effects of the standard chemotherapy drugs in combination with CG200745 at different concentrations. CI < 1, CI = 1, and CI > 1 were indicative of synergistic, additive, and antagonistic effects, respectively. DRI measured the fold reduction in drug dose in a synergistic combination as compared to that of each drug alone, with DRI = 1, DRI > 1, and DRI < 1 indicating no dose reduction and favourable and unfavourable reductions, respectively. In SNU-1196 and SNU-308, CG200745 had both additive and synergistic effects in combination with gemcitabine, cisplatin, 5-FU, and oxaliplatin, as evidenced by the CI and DRI values (Supplementary Tables 1a,b and 2a,b). In SNU-1196, for instance, CG200745 exhibited synergistic cytotoxicity at different gemcitabine concentrations (0.001 to 10.0 μM), with CI values of 0.27 to 0.59 in the presence of 0.25 or 0.5 μM CG200745, respectively (Supplementary Table 1b). Moreover, 0.038 μM of gemcitabine was required to achieve 50% inhibition; however, a 19-fold or lower concentration was required to achieve the same IC_50_ with 0.25 μM CG200745 and 100-fold or lower concentration was required with 0.5 μM CG200745 (Supplementary Table 1a, Dose reduction index). Other drugs also showed synergism and favourable dose reduction with CG200745 (Supplementary Tables 1a,b and 2a,b). Likewise, synergistic concentration-dependent cytotoxicity was observed in triple combinations of gemcitabine plus cisplatin in combination with CG200745 in both cell lines (0.5 μM for SNU-1196 and 1.0 μM for SNU-308) (Fig. 3a, Supplementary Fig. 4e, and Supplementary Tables 1c and 2c). CG200745 was found to reduce the expression of c-MET and increase that of cleaved Caspase-3, which was associated with increased apoptosis (Fig. 3b, and Supplementary Fig. 4f).

### Gemcitabine or 5-FU combined with CG200745 suppresses proliferation of GR cholangiocarcinoma cells

**Figure 4:**
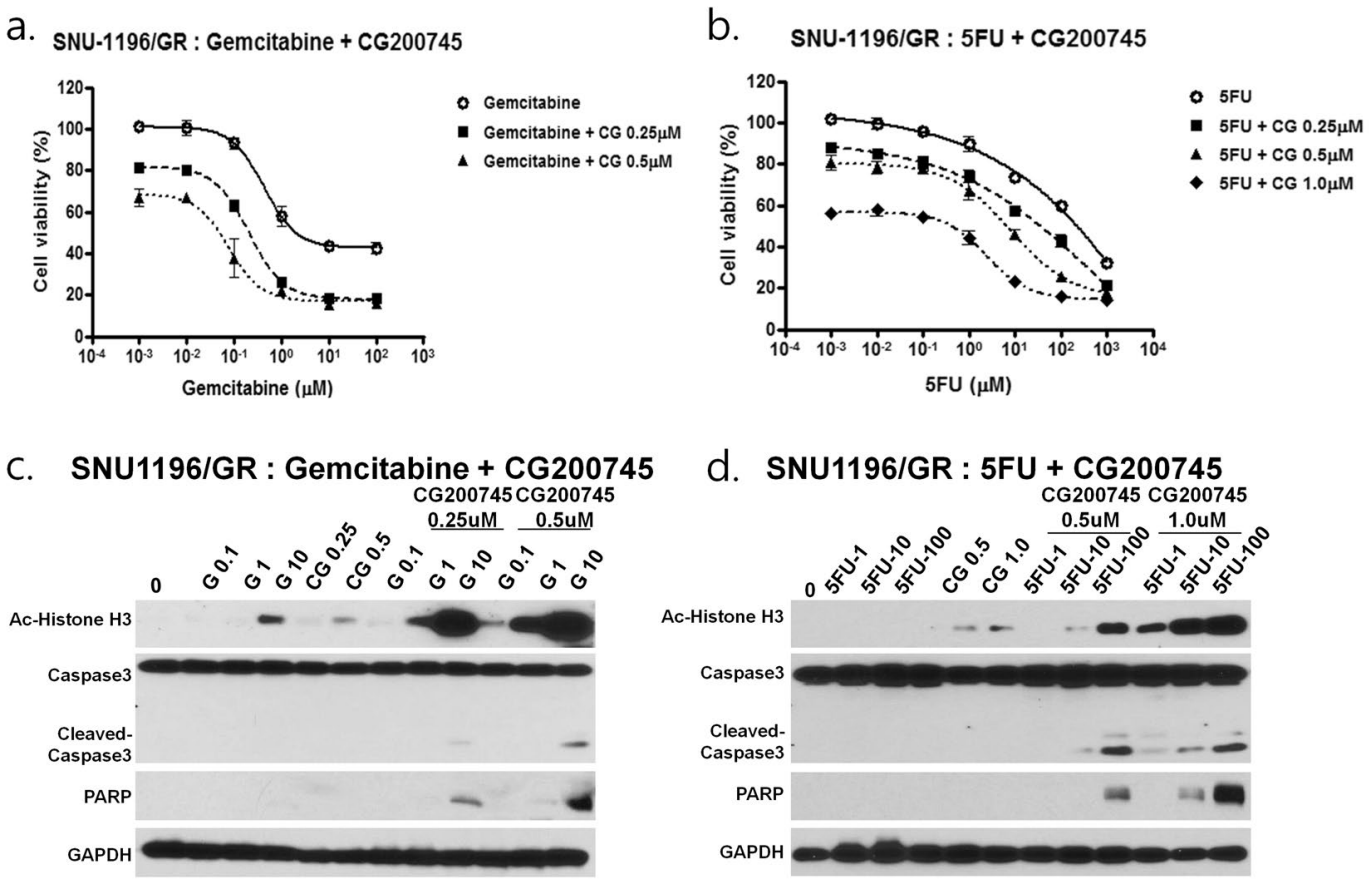
Anti-proliferative effects of gemcitabine and 5-FU combined with CG200745 in SNU-1196/GR. (**a**) SNU-1196/GR cells were treated with gemcitabine with or without CG200745. The IC_50_ of gemcitabine decreased from 2.291 to 0.209 μM at 0.25 μM CG200745 and to 0.048 μM at 0.5 μM CG200745; the value also decreased in SNU-1196/GR cells treated with 5-FU with or without CG200745 from 229.0 to 35.481 μM at 0.25 μM CG200745, to 6.761 μM at 0.5 μM CG200745, and to 0.417 μM at 1.0 μM CG200745. (**c**,**d**) Treatment of SNU-1196/GR cells with CG200745 increased cleaved-Caspase-3 and PARP levels and induced apoptosis

We established the SNU-1196/GR cell line to mimic the case where first-line chemotherapy fails due to the development of drug tolerance. Cells were treated with gemcitabine with or without CG200745. The IC_50_ of gemcitabine decreased from 2.291 to 0.209 and 0.048 μM in the presence of 0.25 and 0.5 μM CG200745, respectively (Fig. 4a). The IC_50_ of 5-FU (229.0 μM) decreased to 35.481, 6.761, and 0.417 μM upon treatment with 0.25, 0.5, and 1.0 μM CG20074, respectively (Fig. 4b). These results suggest a synergistic cytotoxicity (CI < 1) (Supplementary Table 3b). Addition of CG200745 also increased the levels of cleaved Caspase-3 and poly (ADP ribose) polymerase (PARP), which was associated with increased apoptosis (Fig. 4c,d). Thus, CG200745 increased the sensitivity of cells to gemcitabine plus 5-FU in a dose-dependent manner.

### CG200745 suppresses proliferation of cholangiocarcinoma cells in a xenograft model

**Figure 5:**
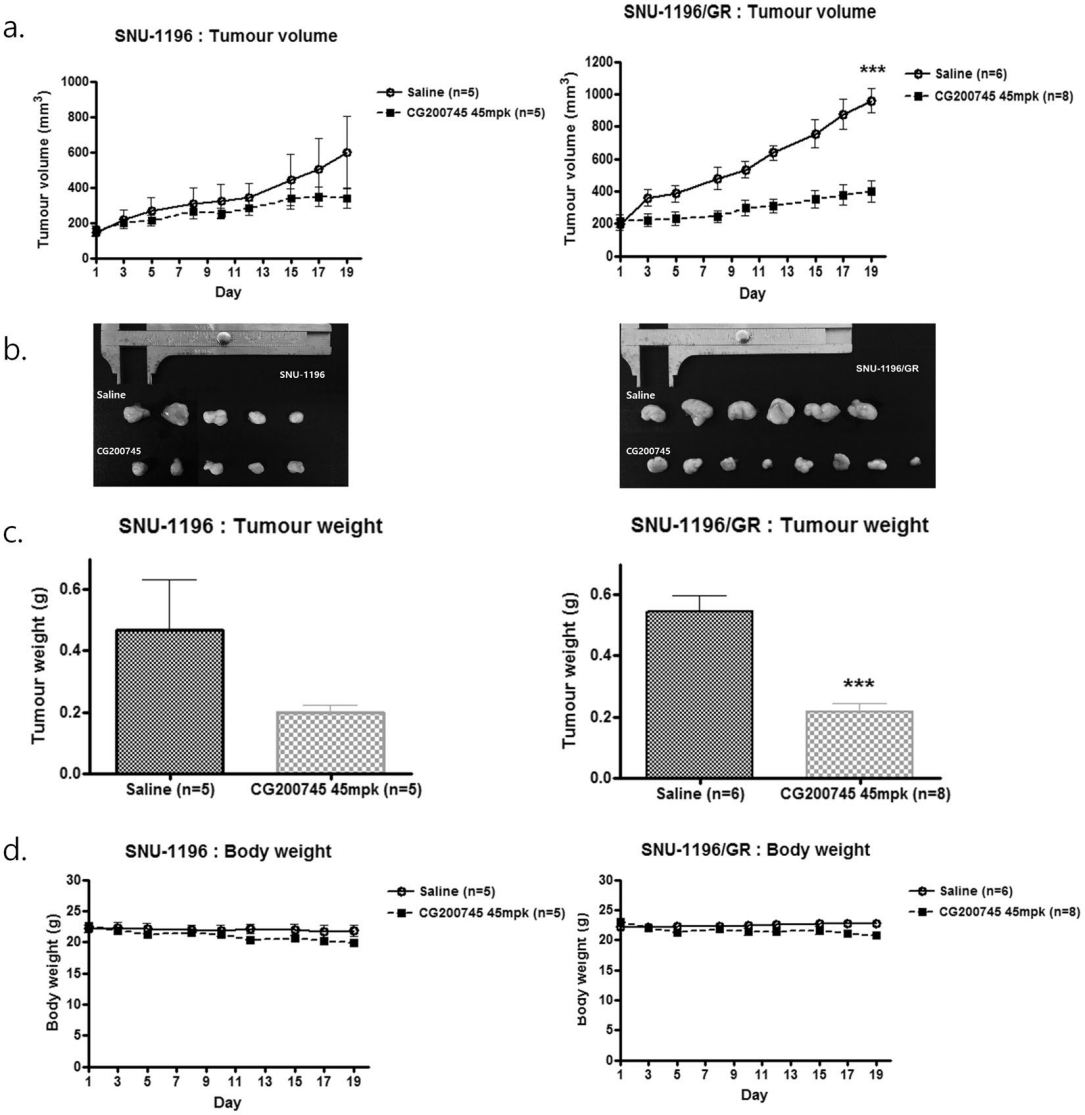
Anti-proliferative activity of CG200745 in cholangiocarcinoma cells in a xenograft model. (**a**,**b**) Tumour size was measured twice weekly and at the end of the experiment, mice were sacrificed and tumours were dissected. Tumour volume was decreased in SNU-1196 and SNU-1196/GR cells treated with CG200745. Average tumour volumes in SNU-1196-injected mice were 599.4 ± 205.1 and 340.6 ± 57.1 mm^3^ (P = 0.06) for the saline and CG200745 groups, respectively; in SNU-1196/GR-injected mice, they were 959.0 ± 73.80 and 398.8 ± 67.42 mm^3^ (P < 0.001) for the saline and CG200745 groups, respectively. Five mice each were used for the SNU-1196 saline and CG200745 groups and six and eight mice were used for the SNU-1196/GR saline and CG200745 groups, respectively. Error bars represent SEM. ***p < 0.001. (**c**) Average tumour weights in SNU-1196-injected mice were 0.469 ± 0.162 and 0.200 ± 0.024 g (P = 0.07) for the saline and CG200745 groups, respectively; in SNU-1196/GR-injected mice, they were 0.543 ± 0.055 and 0.217 ± 0.026 g (P < 0.001) for the saline and CG200745 groups, respectively. Error bars are ± SEM. ***p < 0.001. (**d**) Average body weights of SNU-1196-injected mice were 21.9 ± 0.90 and 19.9 ± 0.55 g for the saline and CG200745 groups, respectively; in SNU-1196/GR-injected mice, they were 22.717 ± 0.342 and 20.737 ± 0.545 g for the saline and CG200745 groups, respectively.

In mice with SNU-1196 and SNU-1196/GR cell-derived tumours, CG200745 treatment decreased tumour volume and body weight relative to saline-treated mice. Average tumour volumes were 340.6 ± 57.1 and 599.4 ± 205.1 mm^3^ for the SNU-1196 group and 398.8 ± 67.42 and 959.0 ± 73.80 mm^3^ for the SNU-1196/GR group injected with CG200745 and saline, respectively (Fig. 5a,b). Average tumour weights were 0.200 ± 0.024 and 0.469 ± 0.162 g for the SNU-1196 group and 0.217 ± 0.026 and 0.543 ± 0.055 g for the SNU-1196/GR group injected with CG200745 and saline, respectively (Fig. 5c). Average body weights were 19.9 ± 0.55 and 21.9 ± 0.90 g for the SNU-1196 group and 20.737 ± 0.545 and 22.717 ± 0.342 g for the SNU-1196/GR group injected with CG200745 and saline, respectively (Fig. 5d). All mice treated with CG200745 showed a < 10% decrease in body weight as compared to saline-treated animals. However, there were no significant differences tumour volume (P = 0.06) or body weight (P = 0.07) between the SNU-1196 groups.

### CG200745 alters gene and miRNA expression profiles in cholangiocarcinoma cells

**Figure 6:**
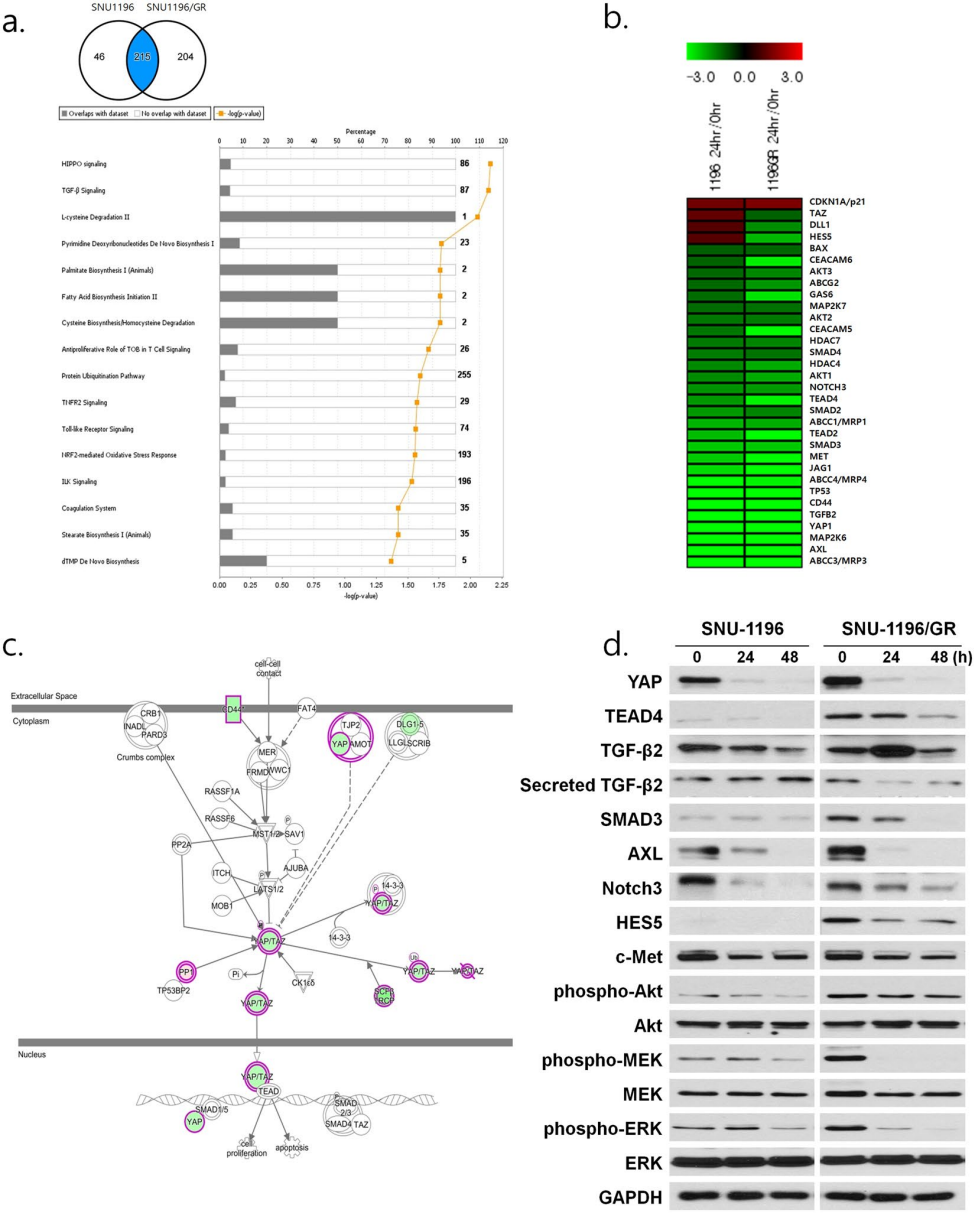
Target gene identification by microarray analysis. (**a**) Gene expression profiles were analysed with Affymetrix Human Genechip 2.0, and 215 differentially expressed genes (cut-off: fold change ≥ 4, P < 1.0E−10) were further examined by IPA. The graph shows the top cellular functions at 24 h post-CG200745 (IC_50_) treatment. (**b**) Genes differentially expressed in response to CG200745 (IC_50_) treatment in SNU-1196 and SNU-1196/GR are shown in the heat map. Up- and downregulated genes are shown in red and green, respectively. (**c**) Top-ranked Hippo signalling-associated genes were downregulated (green). (**d**) Associations among highly ranked pathway-associated genes and corresponding protein levels were analysed following treatment with CG200745 (IC_50_) for 0, 24, and 48 h.

**Figure 7:**
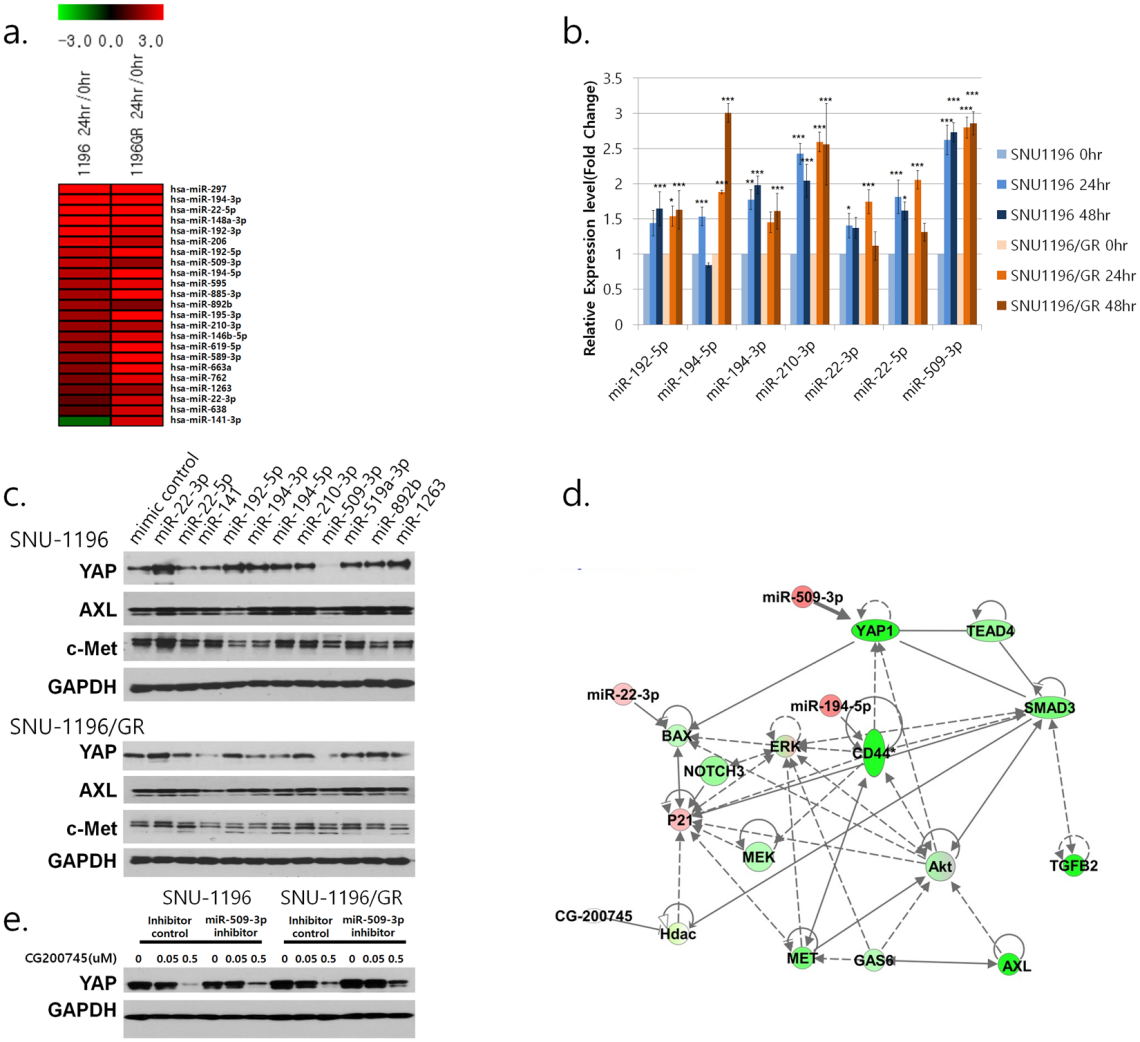
MiRNAs differentially expressed in response to CG200745 treatment and gene pathway analysis. (**a**) MiRNA expression profiles were analysed with a human Affymetrix GeneChip miRNA 4.0 Array and the expression induced by CG200745 treatment is shown in the heat map (upregulated, red). (**b**) Expression of individual miRNAs, as evaluated by qRT-PCR. *P ≤ 0.05, **P < 0.01, and ***P < 0.001. (**c**) SNU-1196 and SNU-1196/GR cells were transfected with miRNA mimics or a mimic control sequence and protein expression post-treatment (24 h) was analysed by western blotting. (**d**) Associations among highly ranked pathway-associated-genes (downregulated, green) and differentially expressed miRNAs (upregulated, red) were analysed following treatment with CG200745 (IC_50_). Solid and dotted lines indicate direct and indirect interactions, respectively. (**e**) SNU-1196 and SNU-1196/GR cells were transfected with miR-509-3p inhibitor or an inhibitor control sequence followed by CG200745 treatment and protein expression post-treatment (48 h) was analysed by western blotting.

The effects of CG200745 on SNU-1196 and SNU-1196/GR cells were assessed by cDNA and miRNA microarray analyses after 24 h of treatment. Complete microarray data on differentially expressed genes (fold change ≥ 2 and P < 0.001) and miRNAs (fold change ≥ 1.5 and P < 0.01) are available in the Array Express database (E-MTAB-5733). The relationships between gene and miRNA expression patterns among CG200745-treated and untreated SNU-1196 and SNU-1196/GR cells were estimated by hierarchical clustering (Supplementary Fig. 5a,b). The overall expression patterns of genes and miRNAs were similar in both cell lines upon treatment with CG200745. A total of 465 differentially expressed genes with a fold change ≥ 4.0 and P value of 0.1 × 10^−10^ were mapped by Ingenuity Pathway Analysis (IPA). There were 215 genes common to both cell lines; canonical pathways were analysed based on the ratio of the number of genes from the dataset that mapped to the pathway and a Fisher’s exact test value. The top statistically significant pathway was Hippo signalling; genes in this pathway including yes-associated protein (*YAP*), tafazzin (*TAZ*), and cluster of differentiation 44 were downregulated upon CG200745 treatment (Fig. 6a–c). Genes that were differentially expressed such as the Hippo pathway-associated transcription factor TEA domain transcription factor (*TEAD*)4; transforming growth factor (TGF)-β signalling-associated genes *TGF-β2* and mothers against decapentaplegic homolog (*SMAD*)3; apoptosis-related genes *AXL* and growth arrest-specific (*GAS*)6; Notch signalling-associated genes *NOTCH3* and Hairy and enhancer of split 5 (*HES5*); the pro-apoptotic gene p21; and proto-oncogenes including c-MET, AKT, mitogen-activated protein kinase, and extracellular signal-regulated kinase were also evaluated by IPA, and protein expression was confirmed by western blotting (Fig. 6d). Microarray and qRT-PCR analyses revealed that miR-22-3p, miR-22-5p, miR-194-5p, miR-194-3p, miR-194-5p, and miR-210-3p were overexpressed in SNU-1196 and SNU-1196/GR cells treated with CG200745 (Fig. 7a,b). Interestingly, miR-509-3p was not expressed in untreated cells, but was induced by the presence of CG200745; the relative expression level was analysed after 12, 24, and 48 h of treatment instead of at 0, 24, and 48 h. The expression of miR-22-3p, miR-192-5p, miR-194-5p, and miR-509-3p was evaluated by IPA and that of the putative targets YAP, AXL, and c-MET was determined in cells transfected with miRNA mimics (Fig. 7c,d). After 24 h, the levels of all three proteins were reduced in cells transfected with miR-509-3p (Fig. 7c).

**Figure 8:**
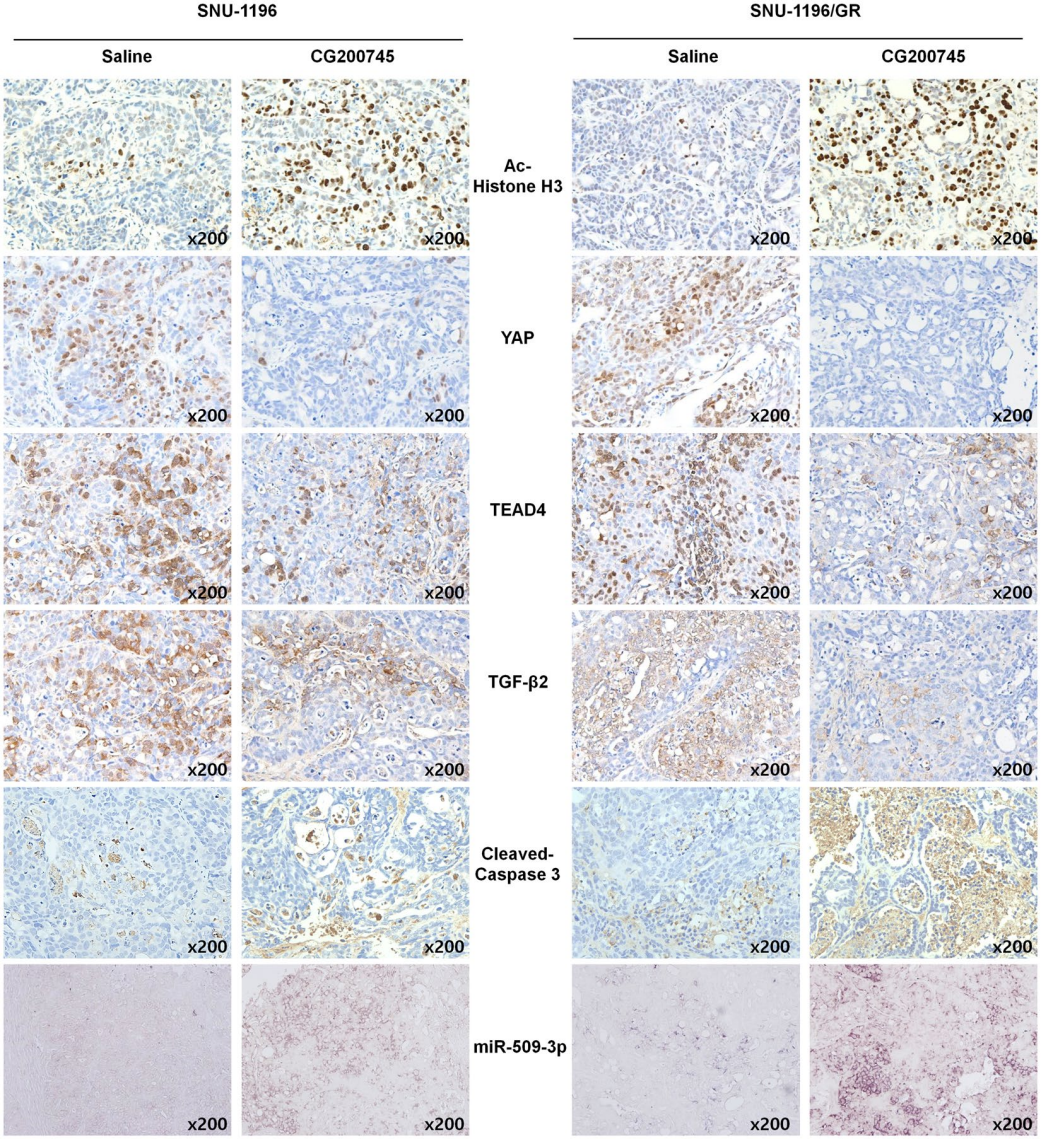
Detection of genes and proteins differentially expressed in response to CG200745 treatment, as detected by *in situ* hybridization and immunohistochemistry, respectively. Tumour tissue was labelled with antibodies against acetylated histone H3, YAP, TEAD4, TGF-β2, and cleaved Caspase 3 and hybridised with an RNA probe targeting miR-509-3p.

To determine whether the increase in miR-509-3p expression induced by CG200745 altered YAP protein level, SNU-1196 and SNU-1196/GR cells were transfected with miR-509-3p inhibitor and miRNA mimics followed by treatment with CG200745 (0.05 and 0.5 μM). Cells transfected with miR-509-3p inhibitor had higher YAP protein levels than those transfected with the control sequence (Fig. 7e). Moreover, Acetylated histone H3 and cleaved-Caspase 3 were upregulated whereas YAP, TEAD4, and TGF-β2 were downregulated in mice treated with CG200745 relative to controls irrespective of the injected cell line. MiR-509-3p expression was also induced by CG200745 treatment (Fig. 8).

## Discussion

HDAC inhibitors are the most widely used anti-cancer drugs targeting epigenetic modifications^9^ to selectively alter gene expression. These compounds suppress the expression of genes involved in tumour progression, invasion, and angiogenesis. Vorinostat and entinostat induce the cyclin-dependent kinase inhibitor p21 to alter expression of proapoptic proteins of the Bcl-2 family^21, 22^, and have therapeutic potential for many types of malignancies including colon cancer, glioma, lung cancer, breast cancer, and hepatocellular carcinoma as a single treatment or in combination with other drugs^23–26^. However, the mechanism underlying the anticancer effects of HDAC inhibitor-induced acetylation is not well understood. Here we demonstrate that a novel HDAC inhibitor, CG200745, selectively targets Hippo signalling in cholangiocarcinoma cells to suppress proliferation and induce apoptosis, acting either alone or synergistically with conventional chemotherapy drugs. Moreover, CG200745 enhanced the sensitivity of gemcitabine-resistant cholangiocarcinoma cells to these drugs.

We recently reported that CG200745 either alone or combined with gemcitabine/erlotinib had synergistic anti-tumour effects in pancreatic cancer cells^20^. In cholangiocarcinoma cells, CG200745 dose-dependently inhibited the proliferation of SNU-1196, SNU-1196/GR, and SNU-308 cells, an effect that was more potent than those of vorinostat and entinostat, which show 2- to 50-fold higher IC_50_ values *in vitro*. In a xenograft model, tumour volume and weight were reduced in both SNU-1196 and SNU-1196/GR groups administered CG200745, with < 10% decreases in body weight. Some HDAC inhibitors are of limited therapeutic use due to toxic side effects at high doses; a lower IC_50_ can reduce these side effects. In human trials, CG200745 showed no toxicity at the tested doses, although a number of patients experienced grade 3 and 4 hematologic toxicity with symptoms such as anaemia and neutropenia that lasted for 1 week, as well as grade 1 and 2 toxicity including mild fatigue and anorexia^27^.

Gemcitabine-based regimens are the first-line chemotherapy for cholangiocarcinoma. Previous studies have shown that combined use of conventional drugs with HDAC inhibitors more potently inhibited cholangiocarcinoma cell proliferation^11, 13, 19, 28^. CG200745 showed synergistic anti-tumour effects with gemcitabine, 5-FU, cisplatin, and oxaliplatin in these cells, while combined treatment of low-dose chemotherapy drugs and CG200745 showed greater inhibition of cell proliferation than either agent alone. Gemcitabine combined with cisplatin is a standard treatment for patients with advanced biliary tract cancer^29^. We found that the triple combination of gemcitabine, cisplatin, and CG200745 (0.5 μM) had both additive and synergistic effects and that the presence of CG200745 decreased IC_50_ by approximately 4-fold relative to treatment with gemcitabine plus cisplatin only.

The above-described effects may be due to CG200745-induced gene expression. The IPA revealed changes in gene expression at the mRNA level following treatment, including downregulation of the Hippo signalling pathway components YAP, TAZ, and TEAD; these have been implicated in cancer development and cancer cell proliferation, invasion, and metastasis, while YAP is frequently hyperactivated in human cancers^30, 31^. TGF-β pathway-associated genes including TGF-β and SMAD and Notch signalling-associated genes were also downregulated in SNU-1196 and SNU-1196/GR cells. A previous study demonstrated that expression of the apoptosis-inducing genes AXL and GAS6 was decreased by CG200745 treatment^32^. Interestingly, we observed that CG200745 altered the expression levels of miR-22-3p, miR-22-5p, miR-194-5p, miR-194-3p, miR-194-5p, miR-210-3p, and miR-509-3p. MiR-509-3p has been reported to act as a tumour suppressor by inhibiting gastric cancer cell proliferation and migration, and its overexpression was shown to induce apoptosis and inhibit breast and lung cancer cell invasion^33, 34^. MiR-509-3p was found to suppress metastasis in breast cancer cells and increase the sensitivity of epithelial ovarian cancer cells to cisplatin-induced apoptosis^35^. It was also recently reported that miR-509-3p directly targets *YAP* and miR-509-3p mimic decreased YAP expression, thereby suppressing migration of ovarian cancer cells^36^. We observed that transient transfection of miR-509-3p mimic decreased YAP expression while miR-509-3p inhibitor abrogated the decrease in YAP expression induced by CG200745.

Gemcitabine-based regimens are routinely used to treat cholangiocarcinoma; however, the development of drug resistance is a major problem. This can be due to enhanced expression of ABC transporters in cancer cells^37, 38^. Increased expression of the human ABC family genes *ABCB*, *ABCC* (which includes MRPs), and *ABCG* confers drug resistance to cancer cells by enhancing drug efflux^39^, while upregulation of *ABCB1*, *ABCC1*, and *ABCG2* is linked to drug resistance in human pancreatic cancer^40^. The sensitivity of human pancreatic cancer to gemcitabine was shown to be dependent on the level of hENT1 expression^41^. The SNU-1196/GR cell line showed an approximately 60-fold increase in tolerance to gemcitabine (IC_50_ = 2.291 μM) without losing sensitivity to CG200745 (IC_50_ = 0.93 μM), and upregulated ABCG2 and MRP4 protein expression *in vitro* and *in vivo* (Supplementary Fig. 1c), which was abrogated by CG200745. The expression of HDAC4, HDAC7, hENT1, and ABC transporters MRP1 and MRP3 in SNU-1196, SNU-1196/GR, and SNU-308 cells was also reduced by CG200745 treatment. HDAC4 and HDAC7 have been implicated in tumour growth, metastasis, and chemosensitivity^42–44^. A decrease in HDAC4 expression may result from upregulation of miRNAs targeting HDAC proteins, given that miR-22-3p was shown to target HDAC4 and that miR-22-3p expression was increased by CG200745 treatment^45, 46^. SNU-1196/GR cells not only exhibited tolerance to gemcitabine but also stem-cell-like features. ALDH is a marker of cancer stem cells (CSCs) and is highly expressed in cholangiocarcinoma cells^47^. The expression of YAP and TEAD—another stem cell feature—was increased in basal breast cancers and was associated with enhanced tumorigenic potential^30, 31^. CSCs contribute to tumour initiation, metastasis, recurrence, and chemotherapy resistance^48^. Resistance to gemcitabine can arise from elevated expression of multidrug-resistance genes or an increase in the number of ALDH-positive cells CSCs; combined use of CG200745 with gemcitabine or 5-FU in SNU-1196/GR cells resulted in more potent inhibition of cell growth. It is worth noting that the IC_50_ of SNU-1196/GR to gemcitabine decreased more than 10 fold with 0.25 μM CG200745 and 45 fold with 0.5 μM CG200745 as compared to gemcitabine alone; the CI and DRI clearly demonstrated both the additive and synergistic effects of gemcitabine and CG200745. Similar trends were observed for 5-FU. However, we only examined the effect of CG200745 on SNU-1196/GR cells; although we attempted to establish GR SNU-308 cells, treatment with increasing doses of gemcitabine increased sensitivity by only 2 fold (Supplementary Fig. 3a).

In conclusion, our study characterised the novel HDAC inhibitor CG200745, which demonstrated anti-tumour effects against cholangiocarcinoma cells *in vitro* and *in vivo* with additive and synergistic effects when used in combination with standard chemotherapy drugs. Given the treatment complications such as drug resistance and toxicity associated with conventional therapies, adding CG200745 to the chemotherapeutic regimen could be a safer treatment for cholangiocarcinoma.

## Methods

### Cell culture

SNU-1196 and SNU-308 human cholangiocarcinoma cell lines were purchased from the Korean Cell Line Bank (Seoul, Korea) and were maintained in RPMI 1640 (Gibco, Grand Island, NY, USA) containing 10% foetal bovine serum (Hyclone, Logan, UT, USA) in a humidified incubator of 5% CO_2_ at 37 °C. GR cell lines were established by treating cells with increasing doses of gemcitabine^49^, and resistance was determined by cell viability analysis. ALDH level was determined by western blotting; and enzymatic activity was measured using the ALDEFLUOR kit (Stem Cell Technologies, Vancouver, BC, Canada) according to the manufacturer’s protocol. Cells treated with the specific ALDH inhibitor diethylminobenzaldehyde was used as a negative control, and ALDH activity was measured by flow cytometry on an LSR II system (BD Biosciences, San Jose, CA, USA).

### Reagents

CG200745 was provided by CrystalGenomics (Seoul, Korea). A 50-mM stock solution was prepared in dimethyl sulfoxide (DMSO, Sigma-Aldrich, St. Louis, MO, USA) and stored at −20 °C until use. HDAC inhibitors and chemotherapy drugs including vorinostat, entinostat, 5-FU, cisplatin, and oxaliplatin were purchased from Selleck Chemicals (Houston, TX, USA); gemcitabine was supplied by Eli Lilly Korea (Seoul, Korea).

### Cell viability assay

Cells were seeded at 3–5 × 10^3^/well in 96-well plates and treated with various compounds at a range of concentrations for 72 h. Cell viability was evaluated with the 3-(4,5-dimethylthiazol-2yl)-2,5-diphenyltetrazolium bromide (MTT) assay (AMRESCO, Solon, OH, USA). Half-maximal inhibitory concentration (IC_50_) was analysed relative to the DMSO control. Values are shown as the means of triplicate wells from three independent experiments for each drug concentration.

### PCR

Total RNA was extracted using an RNeasy Mini kit (Qiagen, Hilden, Germany) and cDNA was synthesised using a SuperScript II kit (Invitrogen, Carlsbad, CA, USA) according to manufacturer’s protocol. β-Actin was used as a reference gene; primers are listed in Supplementary Table 4.

### Microarray analysis

Total RNA containing miRNAs was extracted using a mirVana miRNA Isolation kit (Applied Biosystems, Foster City, CA, USA) according to the manufacturer’s instructions. For the cDNA microarray, cDNA was synthesised using GeneChip Whole Transcript (WT) Amplification kit (Affymetrix, Santa Clara, CA, USA) according to the manufacturer’s instructions; sense cDNA was fragmented and biotin-labelled with terminal deoxynucleotidyl transferase using the GeneChip WT Terminal labelling kit (Affymetrix). Approximately 5.5 μg of labelled target DNA was hybridised to the Affymetrix GeneChip Human Gene 2.0 ST Array at 45 °C for 16 h; hybridised arrays were washed and stained on a GeneChip Fluidics Station 450 and scanned on a GCS3000 instrument (Affymetrix). Export, processing, and analysis of array data were carried out using Affymetrix GeneChip Command Console (AGCC), Affymetrix Expression Console, and R 3.0.2 (www.r-project.org) software. For the miRNA microarray, 1 μg of total RNA was biotin-labelled with the FlashTag Biotin HSR RNA Labelling kit (Affymetrix) and labelled samples were hybridised to a human Affymetrix GeneChip miRNA 4.0 Array (*Homo sapiens*) using the GeneChip Hybridization Oven according to the manufacturer’s protocols. Arrays were scanned using the GeneChip Scanner and array data export, processing, and analysis were carried out using AGCC. Both cDNA and miRNA analyses were performed twice with independent sample sets. Pathway enrichment analysis was performed with Ingenuity Pathway Analysis (IPA) software (Qiagen), and differentially expressed genes were visualised with the MeV microarray analysis platform (www.tm4.org/mev.html).

### Western blotting

Cells were lysed in a buffer composed of 70 mM glycerophosphate (pH 7.2), 0.6 mM Na vanadate, 2 mM MgCl_2_, 1 mM EGTA, 1 mM dithiothreitol, 0.5% Triton X-100, 0.2 mM phenylmethylsulfonyl fluoride, and 1 × complete protease inhibitor (Roche Applied Science, Nutley, NJ, USA). Proteins (25 μg) were separated on sodium dodecyl sulphate-polyacrylamide gels and transferred to a polyvinylidene difluoride membrane (Immobilon-P; Millipore, Bedford, MA, USA) that was blocked in 5% (w/v) non-fat dry milk (Bio-Rad Laboratories, Hercules, CA, USA) and incubated overnight at 4 °C with antibodies diluted 1:1000. Horseradish peroxidase-conjugated secondary antibody was used for detection and immunoblots were developed with West Pico Chemiluminescent substrate (Thermo Fisher Scientific, Waltham, MA, USA). Antibodies used for western blot analysis are listed in Supplementary Table 5.

### MiRNA quantitative reverse transcription (qRT-)PCR

qRT-PCR was carried out with the TaqMan Advanced miRNA cDNA Synthesis kit (Applied Biosystems), TaqMan Advanced miRNA Assay (Applied Biosystems), and TaqMan Gene Expression Master Mix according to the manufacturers’ protocols on an ABI Prism 7300 Sequence Detection System (Applied Biosystems). Primers for mature miRNAs were purchased from Applied Biosystems. Standard curves were generated and relative amounts of target miRNAs were normalised to that of miR-331-3p. Relative quantification of miRNAs within samples were calculated using the comparative Ct method (ΔCt_post-treatment_ − ΔCt_pre-treatment_ = ΔΔCt; relative quantity = 2^−ΔΔCt^) and converted to fold changes. qRT-PCR reactions were prepared in triplicate from three different total RNA samples.

### Transient transfection

Cells were transfected with pre-made miRNA mimics, mimic control sequence, miRNA inhibitors or inhibitor control sequence (all from Bioneer, Daejun, Korea) at a final concentration of 20 nM using RNAiMAX (Invitrogen) in Opti-MEM (Gibco).

### *In vivo* effects of CG200745

The study protocol was approved by the ethics committee of the Department of Laboratory Animal Resources, Yonsei Biomedical Research Institute, Yonsei University College of Medicine (2015-0205). Experiments were carried out using 6-week-old male BALB/c nude mice (Japan SLC, Yokohama, Japan), and were approved by the Institutional Animal Care and Use Committee of Yonsei University College of Medicine based on the Animal Protection Act. SNU-1196 and SNU-1196/GR cells in exponential phase (5 × 10^6^/mouse) were subcutaneously injected into the right flank. Drug treatment was initiated 2 weeks after tumour implantation when tumours had reached a volume of approximately 200 mm^3^. CG200745 (45 mg/kg) or saline was delivered into the peritoneum for 3 weeks (on days 1, 3, 5, 7, 9, 11, 13, 15, 17, and 19). Tumour formation was monitored three times a week by measuring the width and length, and tumour volume was calculated using the formula: volume (mm^3^) = (length × width^2^)/2. Animals were sacrificed 3 weeks after the first administration.

### Immunohistochemistry and in situ hybridization

Mouse tumour specimens were embedded in paraffin and stained with hematoxylin and eosin according to standard protocols. For immunohistochemistry, sections of paraffin-embedded tissue were deparaffinised in xylene and rehydrated in a graded ethanol series. Endogenous peroxidase activity was blocked by incubation in methanol containing 0.3% hydrogen peroxide at room temperature for 20 min. Antigen retrieval was performed in citrate buffer (0.01 M, pH 6.0), followed by blocking in 10% normal donkey serum for 1 h at room temperature. Slides were incubated in primary antibody diluted 1:200 in antibody diluent (Gibco) at 4 °C; antibodies are listed in Supplementary Table 5. The reaction was carried out using an Envision kit (Dako, Carpinteria, CA, USA). Sections were counterstained with hematoxylin. *In situ* hybridization was performed using Exiqon 5′-digoxigenin (DIG)-labelled miRCURY Locked Nucleic Acid (LNA) Detection probes to detect U6, scrambled miRNA, and hsa-miR-509-3p LNA probes. Deparaffinised and rehydrated tissue sections were treated with Proteinase K (20 μg/ml) at 37 °C for 10 min followed by paraformaldehyde (4%) fixation for 5 min. DIG-labelled probes were denatured at 90 °C for 5 min. Tissue was hybridised with probes (10 pmol/μl) and incubated at 53 °C overnight, and washed under stringent conditions at 53 °C. Hybridised probes were then detected with an anti-DIG antibody and visualised by incubation with NBT/BCIP alkaline phosphatase substrate solution (Roche Diagnostics, Indianapolis, IN, USA). Sections were counterstained with Neutral Red and imaged under a BX51 microscope (Olympus, Tokyo, Japan).

### Drug combination studies

The effect of CG200745 combined with gemcitabine, cisplatin, 5-FU, oxaliplatin, and gemcitabine plus cisplatin on cancer cell viability was evaluated in SNU-1196, SNU-1196/GR, and SNU-308 cells using the fixed-ratio method. Cells were treated with CG200745, gemcitabine, cisplatin, 5-FU, and oxaliplatin individually or in combination. The combination treatment consisted of 10-fold serial dilutions of gemcitabine and 5-FU and 5-fold dilutions of cisplatin and oxaliplatin with 0.25 or 0.5 μM CG200745. For triple combination treatment, gemcitabine and cisplatin were combined at a concentration ratio of 1:1 (gemcitabine IC_50_:cisplatin IC_50_ and 2-fold serial dilutions were prepared with 0.25 or 0.5 μM CG200745. After treatment, cell viability was determined with the MTT assay and dose-effect data for individual drugs and their combinations were analysed for synergism using CompuSyn software (http://www.combosyn.com/). CI values were calculated to characterise the nature of the drug interaction as defined by Chou and Talalay: CI =  1, additivity; CI  < 1, synergism; CI > 1, antagonism^50^. The DRI is a measure of the extent to which the dose of a drug in a synergistic combination must be reduced relative to the dose of the same drug alone to achieve a given effect level. The DRI value for each drug was also calculated.

### Statistics

Data were analysed using SPSS v.11.0 (SPSS Inc., Chicago, IL, USA) or GraphPad Prism v.5.0 (GraphPad Inc., La Jolla, CA, USA) software. Values for cell viability are expressed as the mean ± standard deviation and values for the xenograft model are expressed as the mean ± standard error of the mean. Differences between groups were analysed with the t test, and P ≤ 0.05 was considered significant. Asterisks (*, **, and ***) indicate significance at P ≤ 0.05, P < 0.01, and P < 0.001, respectively.

### Data availability

Cell line expression data are available in the ArrayExpress database (http://www.ebi.ac.uk/arrayexpress) under accession number E-MTAB-5733.

## Electronic supplementary material

Supplementary figures and tables

## References

[1] Anderson CD, Pinson CW, Berlin J, Chari RS (2004). Diagnosis and treatment of cholangiocarcinoma. Oncologist.

[2] Wijaya I, Abdullah M (2011). Diagnosis and treatment update: cholangiocarcinoma. Acta Med Indones.

[3] Khan SA (2012). Guidelines for the diagnosis and treatment of cholangiocarcinoma: an update. Gut.

[4] Okusaka T (2010). Gemcitabine alone or in combination with cisplatin in patients with biliary tract cancer: a comparative multicentre study in Japan. Br J Cancer.

[5] Valle J (2010). Cisplatin plus gemcitabine versus gemcitabine for biliary tract cancer. N Engl J Med.

[6] Woo SM (2013). Gemcitabine plus cisplatin versus capecitabine plus cisplatin as first-line chemotherapy for advanced biliary tract cancer: a retrospective cohort study. Chemotherapy.

[7] Legube G, Trouche D (2003). Regulating histone acetyltransferases and deacetylases. EMBO Rep.

[8] Chun SM (2015). Epigenetic modulation with HDAC inhibitor CG200745 induces anti-proliferation in non-small cell lung cancer cells. PLoS One.

[9] Emanuele S, Lauricella M, Tesoriere G (2008). Histone deacetylase inhibitors: apoptotic effects and clinical implications (Review). Int J Oncol.

[10] Duvic M, Dimopoulos M (2016). The safety profile of vorinostat (suberoylanilide hydroxamic acid) in hematologic malignancies: A review of clinical studies. Cancer Treat Rev.

[11] Baradari V, Hopfner M, Huether A, Schuppan D, Scherubl H (2007). Histone deacetylase inhibitor MS-275 alone or combined with bortezomib or sorafenib exhibits strong antiproliferative action in human cholangiocarcinoma cells. World J Gastroenterol.

[12] Xu LN, Wang X, Zou SQ (2008). Effect of histone deacetylase inhibitor on proliferation of biliary tract cancer cell lines. World J Gastroenterol.

[13] Kitamura T (2012). The therapeutic effect of histone deacetylase inhibitor PCI-24781 on gallbladder carcinoma in BK5.erbB2 mice. J Hepatol.

[14] Guo R (2012). MicroRNA miR-491-5p targeting both TP53 and Bcl-XL induces cell apoptosis in SW1990 pancreatic cancer cells through mitochondria mediated pathway. Molecules.

[15] Liu N (2015). The Roles of MicroRNA-122 Overexpression in Inhibiting Proliferation and Invasion and Stimulating Apoptosis of Human Cholangiocarcinoma Cells. Sci Rep.

[16] Scott GK, Mattie MD, Berger CE, Benz SC, Benz CC (2006). Rapid alteration of microRNA levels by histone deacetylase inhibition. Cancer Res.

[17] Bartel DP (2009). MicroRNAs: target recognition and regulatory functions. Cell.

[18] Oh ET (2012). Novel histone deacetylase inhibitor CG200745 induces clonogenic cell death by modulating acetylation of p53 in cancer cells. Invest New Drugs.

[19] Iwahashi S (2011). Effect of histone deacetylase inhibitor in combination with 5-fluorouracil on pancreas cancer and cholangiocarcinoma cell lines. J Med Invest.

[20] Lee HS (2017). A novel HDAC inhibitor, CG200745, inhibits pancreatic cancer cell growth and overcomes gemcitabine resistance. Sci Rep.

[21] Munshi A (2006). Vorinostat, a histone deacetylase inhibitor, enhances the response of human tumor cells to ionizing radiation through prolongation of gamma-H2AX foci. Mol Cancer Ther.

[22] Zhang C, Richon V, Ni X, Talpur R, Duvic M (2005). Selective induction of apoptosis by histone deacetylase inhibitor SAHA in cutaneous T-cell lymphoma cells: relevance to mechanism of therapeutic action. J Invest Dermatol.

[23] Jin JS, Tsao TY, Sun PC, Yu CP, Tzao C (2012). SAHA inhibits the growth of colon tumors by decreasing histone deacetylase and the expression of cyclin D1 and survivin. Pathol Oncol Res.

[24] Chinnaiyan P (2012). Phase I trial of vorinostat combined with bevacizumab and CPT-11 in recurrent glioblastoma. Neuro Oncol.

[25] Ramaswamy B (2012). Phase I-II study of vorinostat plus paclitaxel and bevacizumab in metastatic breast cancer: evidence for vorinostat-induced tubulin acetylation and Hsp90 inhibition *in vivo*. Breast Cancer Res Treat.

[26] Liu YL (2010). Autophagy potentiates the anti-cancer effects of the histone deacetylase inhibitors in hepatocellular carcinoma. Autophagy.

[27] Kim KP (2015). First-in-human study of the toxicity, pharmacokinetics, and pharmacodynamics of CG200745, a pan-HDAC inhibitor, in patients with refractory solid malignancies. Invest New Drugs.

[28] Asgar, M. A., Senawong, G., Sripa, B. & Senawong, T. Synergistic anticancer effects of cisplatin and histone deacetylase inhibitors (SAHA and TSA) on cholangiocarcinoma cell lines. *Int J Oncol* **48**, 409–420 (2016). 10.3892/ijo.2015.3240 26575528

[29] Lee KJ (2016). A pilot study of concurrent chemoradiotherapy with gemcitabine and cisplatin in patients with locally advanced biliary tract cancer. Cancer Chemother Pharmacol.

[30] Harvey KF, Zhang X, Thomas DM (2013). The Hippo pathway and human cancer. Nat Rev Cancer.

[31] Zhou GX (2013). Effects of the hippo signaling pathway in human gastric cancer. Asian Pac J Cancer Prev.

[32] Han J (2013). Gas6/Axl mediates tumor cell apoptosis, migration and invasion and predicts the clinical outcome of osteosarcoma patients. Biochem Biophys Res Commun.

[33] Zhang G, Liu Z, Han Y, Wang X, Yang Z (2016). Overexpression of miR-509 Increases Apoptosis and Inhibits Invasion via Suppression of Tumor Necrosis Factor-alpha in Triple-Negative Breast Cancer Hs578T Cells. Oncol Res.

[34] Wang XH (2016). MiR-509-3-5p causes aberrant mitosis and anti-proliferative effect by suppression of PLK1 in human lung cancer A549 cells. Biochem Biophys Res Commun.

[35] Chen W (2016). MicroRNA-509-3p increases the sensitivity of epithelial ovarian cancer cells to cisplatin-induced apoptosis. Pharmacogenomics.

[36] Pan Y (2016). miR-509-3p is clinically significant and strongly attenuates cellular migration and multi-cellular spheroids in ovarian cancer. Oncotarget.

[37] Marin, J. J. *et al*. Molecular Bases Of Chemoresistance In Cholangiocarcinoma. *Curr Drug Targets* (2015). 10.2174/1389450116666150223121508 25706108

[38] Tepsiri N (2005). Drug sensitivity and drug resistance profiles of human intrahepatic cholangiocarcinoma cell lines. World J Gastroenterol.

[39] Horsey AJ, Cox MH, Sarwat S, Kerr ID (2016). The multidrug transporter ABCG2: still more questions than answers. Biochem Soc Trans.

[40] Pang L (2014). ATP-Binding Cassette Genes Genotype and Expression: A Potential Association with Pancreatic Cancer Development and Chemoresistance?. Gastroenterol Res Pract.

[41] Greenhalf W (2014). Pancreatic cancer hENT1 expression and survival from gemcitabine in patients from the ESPAC-3 trial. J Natl Cancer Inst.

[42] Parra M (2015). Class IIa HDACs - new insights into their functions in physiology and pathology. FEBS J.

[43] Clocchiatti A (2013). Class IIa HDACs repressive activities on MEF2-depedent transcription are associated with poor prognosis of ER(+) breast tumors. FASEB J.

[44] Clocchiatti A, Florean C, Brancolini C (2011). Class IIa HDACs: from important roles in differentiation to possible implications in tumourigenesis. J Cell Mol Med.

[45] Zhang J (2010). microRNA-22, downregulated in hepatocellular carcinoma and correlated with prognosis, suppresses cell proliferation and tumourigenicity. Br J Cancer.

[46] Lu W (2015). The microRNA miR-22 inhibits the histone deacetylase HDAC4 to promote T(H)17 cell-dependent emphysema. Nat Immunol.

[47] Shuang ZY (2014). Transforming growth factor-beta1-induced epithelial-mesenchymal transition generates ALDH-positive cells with stem cell properties in cholangiocarcinoma. Cancer Lett.

[48] Sun S, Wang Z (2010). ALDH high adenoid cystic carcinoma cells display cancer stem cell properties and are responsible for mediating metastasis. Biochem Biophys Res Commun.

[49] Hong SP, Wen J, Bang S, Park S, Song SY (2009). CD44-positive cells are responsible for gemcitabine resistance in pancreatic cancer cells. Int J Cancer.

[50] Chou TC, Talalay P (1984). Quantitative analysis of dose-effect relationships: the combined effects of multiple drugs or enzyme inhibitors. Adv Enzyme Regul.

