# Supplementary material for: CG200745, an HDAC inhibitor, induces anti-tumour effects in cholangiocarcinoma cell lines via miRNAs targeting the Hippo pathway
Source: Sci Rep. 2017 Sep 7;7:10921. doi: 10.1038/s41598-017-11094-3 (PMC5589721; doi:10.1038/s41598-017-11094-3)
Supplement: Supplementary file 1 — Supplementary figures and tables [file 41598_2017_11094_MOESM1_ESM.pdf]

CG200745, an HDAC inhibitor, induces anti-tumour effects in cholangiocarcinoma cell lines via miRNAs targeting the Hippo pathway

Dawoon E. Jung[1]*, Soo Been Park[2]*, Kahee Kim[3], Chanyang Kim[3], and Si Young Song[1, 2, 3†]

*Equal contribution.

[1]Institute of Gastroenterology, Yonsei University College of Medicine, Seoul, Korea

[2]Division of Gastroenterology, Department of Internal Medicine, Yonsei University College of Medicine, Seoul, Korea

[3]Brain Korea 21 PLUS Project for Medical Science, Yonsei University College of Medicine, Seoul, Korea

[†]**Corresponding author:** Si Young Song, MD, PhD. Division of Gastroenterology, Department of Internal Medicine, Yonsei University College of Medicine, 50-1 Yonsei-ro, Seodaemun-gu, Seoul 120-752, Korea. Phone: +82-2-2228-1957, fax: +82-2-2227-7900, e-mail: sysong@yuhs.ac

Supplementary Figure 1. SNU-1196 cells were treated with increasing doses of gemcitabine to establish a GR cell line (SNU-1196/GR). (a, b) Cell viability was assessed with the MTT assay; gemcitabine IC_{50} values were 0.038 and 2.291 µM, respectively. (c) Elevated protein levels of c-MET, phospho-AKT, N-cadherin, and ALDH were analysed by western blotting. (d) ALDH enzymatic activity was measured with the ALDEFLUOR kit; cells treated with the specific ALDH inhibitor diethylminobenzaldehyde served as a negative control. ALDH activity was also measured by flow cytometry.

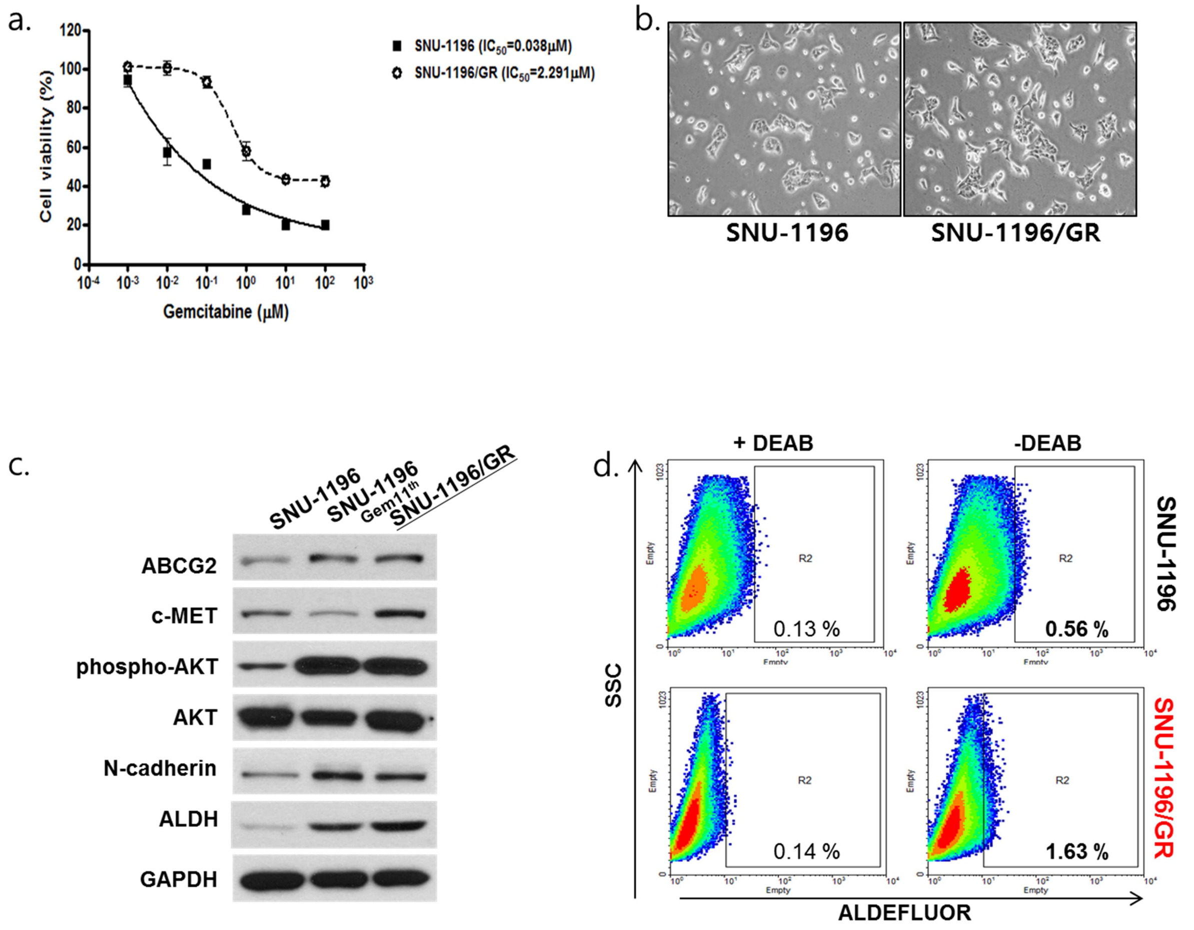

Supplementary Figure 2. Detection of proteins differentially expressed in tumours derived from SNU-1196 and SNU-1196/GR cells, as detected by immunohistochemistry using antibodies against ALDH, ABCG2, and MRP4.

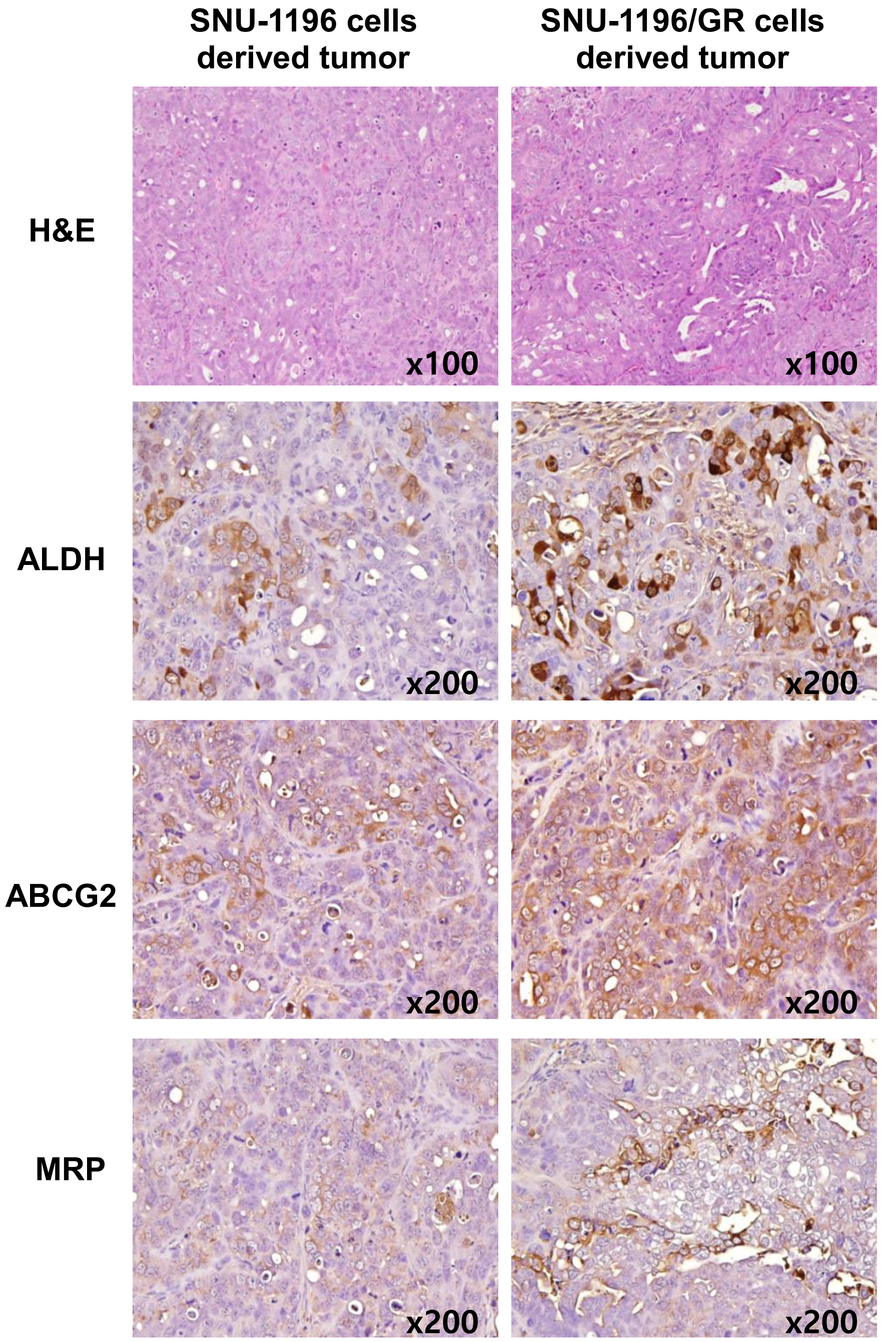

Supplementary Figure 3. Establishment of a GR cell line. (a, b) SNU-308 was treated with increasing doses of gemcitabine to establish a GR cell line (SNU-308/GR). Cell viability was assessed with the MTT assay; gemcitabine IC_{50} values were 2.454 and 5.128 μM, respectively. (c–e) CG200745 (IC_{50}) induced the expression of apoptosis-related protein p21 and inhibited that of c-MET, AKT, phospho-MEK, and phospho-ERK, as determined by western blotting (c); inhibited the expression of multidrug resistance genes, as determined by PCR (d); and altered the expression level of HDAC isozyme, as determined by western blotting (e).

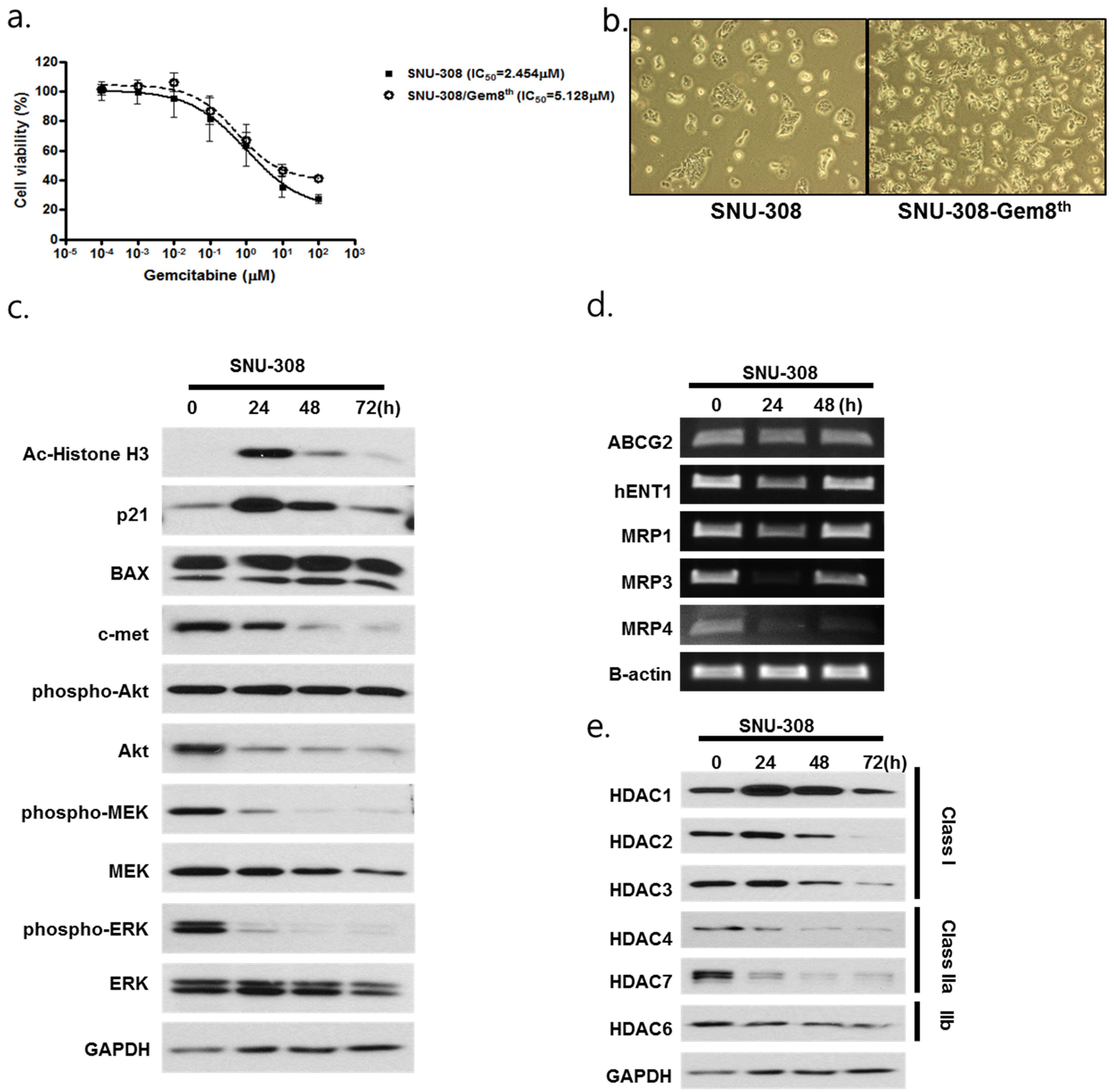

Supplementary Figure 4. Anti-proliferative effects of gemcitabine, cisplatin, 5-FU, and oxaliplatin in combination with CG200745. SNU-308 cells were treated with (a) gemcitabine, (b) cisplatin, (c) 5-FU, and (d) oxaliplatin along with CG200745 and viability was assessed with the MTT assay. *P ≤ 0.05, **P < 001, ***P < 0.001. (e) SNU-308 cells were treated with indicated concentrations of gemcitabine plus cisplatin along with CG200745 and viability was assessed. (f) Cells treated with gemcitabine plus cisplatin and CG200745 showed downregulation of c-MET and upregulation of p21 and cleaved caspase-3.

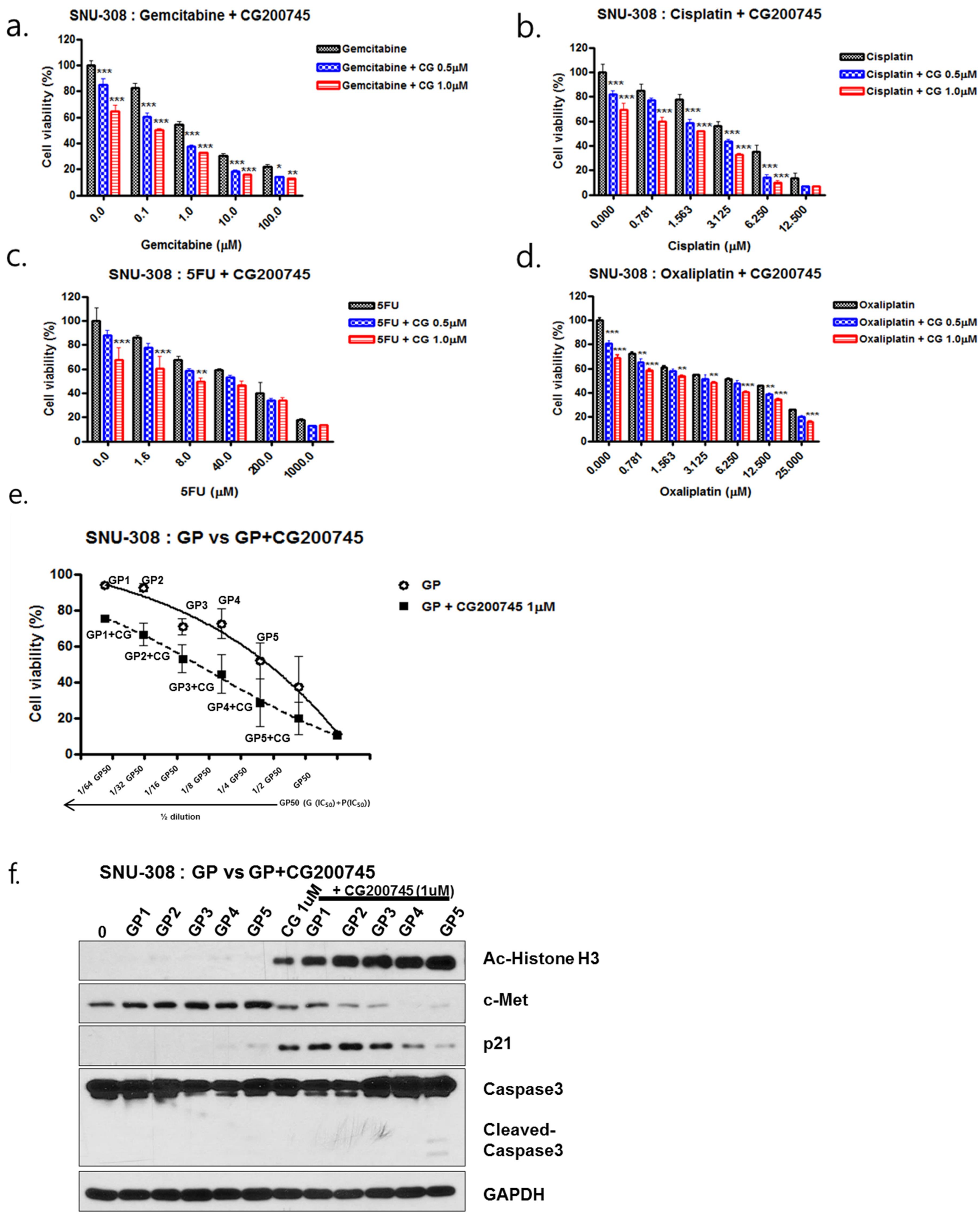

Supplementary Figure 5. (a) Gene expression profiles were analysed using Affymetrix Human Genechip 2.0 and differentially expressed genes (cut-off, fold change ≥ 2, P < 0.05) were identified by cluster analysis-hierarchical clustering (Euclidean method, complete linkage). Based on clustering results, overall gene expression patterns in SNU-1196 and SNU-1196/GR either pre- (0 h) or post- (24 h) treatment with CG200745 were hierarchically organised. (b) MiRNA expression profiles were analysed with a human Affymetrix GeneChip miRNA 4.0 Array and differentially expressed miRNAs (cut-off, fold change ≥ 1.5, P < 0.05) were identified by cluster analysis-hierarchical clustering (Euclidean method, complete linkage). Based on clustering results, overall miRNA expression patterns in SNU-1196 and SNU-1196/GR either pre- (0 h) or post- (24 h) treatment with CG200745 were hierarchically organised.

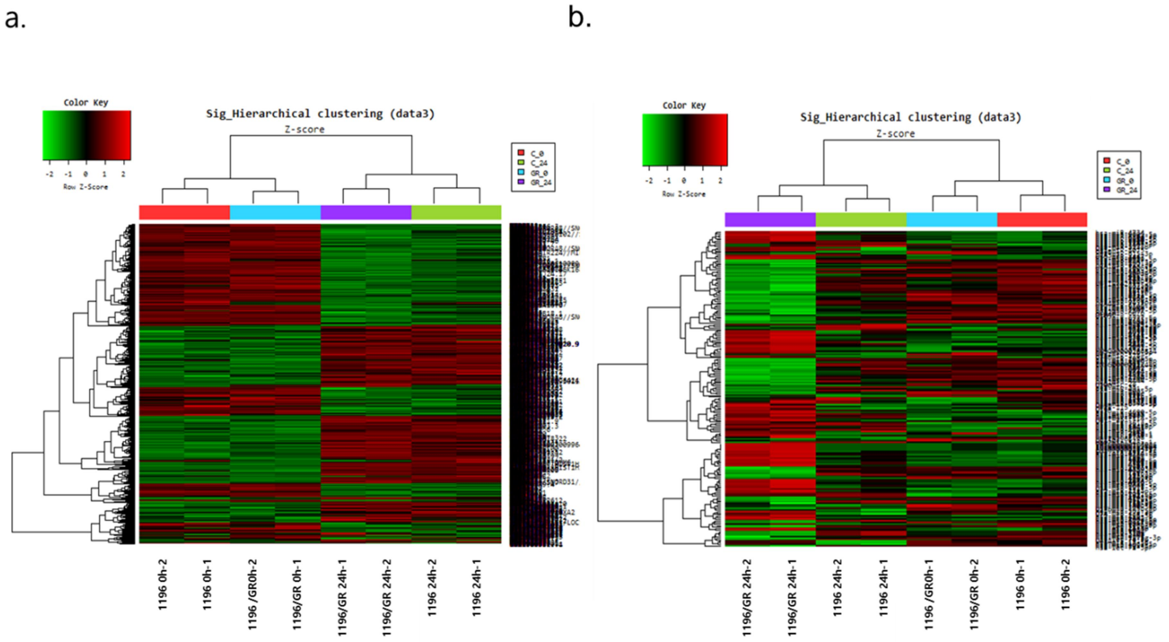

Supplementary Table 1. Combination index (CI) and dose reduction index (DRI) of CG200745 in SNU-1196 cells

a.

**SNU-1196**	**Gemcitabine (μM)**		**Cisplatin (μM)**		**5-FU (μM)**		**Oxaliplatin (μM)**	
Single (IC_{50})	0.038		4.90		104.713		3.236	
**CG200745 (μM)**	0.25	0.50	0.25	0.50	0.25	0.50	0.25	0.50
Combi (IC_{50})	0.002	<0.0001	0.56	0.11	1.86	0.01	1.59	0.25
**Dose reduction index**	19	>100	8.75	44.55	56.29	>100	2.04	12.94

b.

Gemcitabine and CG200745

**Combination Index**	**0.489**	**0.281**	**0.273**	**0.281**	**0.341**
Gemcitabine(μM)	0.001	0.010	0.100	1.000	10.000
CG200745(μM)	0.250	0.250	0.250	0.250	0.250
**Combination Index**	**0.570**	**0.518**	**0.444**	**0.508**	**0.594**
Gemcitabine(μM)	0.001	0.010	0.100	1.000	10.000
CG200745(μM)	0.500	0.500	0.500	0.500	0.500

Cisplatin and CG200745

**Combination Index**	**0.567**	**0.447**	**0.635**
Cisplatin(μM)	0.400	2.000	10.000
CG200745(μM)	0.250	0.250	0.250
**Combination Index**	**1.039**	**0.591**	**0.946**
Cisplatin(μM)	0.400	2.000	10.000
CG200745(μM)	0.500	0.500	0.500

5-FU and CG200745

**Combination Index**	**0.627**	**0.454**	**0.342**	**0.432**	**0.770**
5-FU(μM)	0.100	1.000	10.000	100.000	1000.000
CG200745(μM)	0.250	0.250	0.250	0.250	0.250
**Combination Index**	**0.665**	**0.494**	**0.449**	**0.424**	**0.718**
5-FU(μM)	0.100	1.000	10.000	100.000	1000.000
CG200745(μM)	0.500	0.500	0.500	0.500	0.500

Oxaliplatin and CG200745

**Combination Index**	**0.84091**	**0.92858**	**0.78218**	**0.77119**
Oxaliplatin(μM)	0.080	0.400	2.000	10.000
CG200745(μM)	0.250	0.250	0.250	0.250
**Combination Index**	**0.72283**	**0.62079**	**0.63581**	**0.77325**
Oxaliplatin(μM)	0.080	0.400	2.000	10.000
CG200745(μM)	0.500	0.500	0.500	0.500

c.

Gemcitabine plus Cisplatin and CG200745

**Combination Index**	**0.763**	**0.583**	**0.494**	**0.468**	**0.545**	**0.654**
Gemcitabine(μM)	0.00313	0.00625	0.01250	0.02500	0.05000	0.10000
Cisplatin(μM)	0.12813	0.25625	0.51240	1.02500	2.05000	4.10000
CG200745(μM)	0.50000	0.50000	0.50000	0.50000	0.50000	0.50000

CI>1 indicates angatonism; CI=1 indicates an additive effect; and CI<1 indicates synergism. DRI>1 is favorable dose reduction that lead to toxicity reduction.

Supplementary Table 2. Combination index (CI) and dose reduction index (DRI) of CG200745 in SNU-308 cells

a.

**SNU-308**	**Gemcitabine (μM)**		**Cisplatin (μM)**		**5-FU (μM)**		**Oxaliplatin (μM)**	
Single (IC_{50})	1.413		3.80		74.131		6.918	
**CG200745 (μM)**	0.50	1.00	0.50	1.00	0.50	1.00	0.50	1.00
Combi (IC_{50})	0.32	0.13	2.34	1.82	44.67	17.78	4.57	2.82
**Dose reduction index**	4.47	11.21	1.62	2.09	1.66	4.17	1.51	2.45

b.

Gemcitabine and CG200745

**Combination Index**	**0.559**	**0.369**	**0.244**	**0.640**
Gemcitabine(μM)	0.001	0.010	0.100	1.000
CG200745(μM)	0.500	0.500	0.500	0.500
**Combination Index**	**0.723**	**0.507**	**0.312**	**0.614**
Gemcitabine(μM)	0.001	0.010	0.100	1.000
CG200745(μM)	1.000	1.000	1.000	1.000

Cisplatin and CG200745

**Combination Index**	1.474	1.005	1.001	**0.484**	**0.475**
Cisplatin(μM)	0.001	0.010	0.100	1.000	10.000
CG200745(μM)	0.500	0.500	0.500	0.500	0.500
**Combination Index**	1.016	1.381	**0.772**	**0.366**	**0.484**
Cisplatin(μM)	0.001	0.010	0.100	1.000	10.000
CG200745(μM)	1.000	1.000	1.000	1.000	1.000

5-FU and CG200745

**Combination Index**	**0.892**	**0.772**	1.096	1.313	**0.589**
5-FU(μM)	0.001	0.010	0.100	1.000	10.000
CG200745(μM)	1.000	1.000	1.000	1.000	1.000

Oxaliplatin and CG200745

**Combination Index**	**0.907**	**0.914**	**0.825**	**0.887**	**0.233**
Oxaliplatin(μM)	0.010	0.100	1.000	10.000	10.000
CG200745(μM)	1.000	1.000	1.000	1.000	1.000

c.

Gemcitabine plus Cisplatin and CG200745

**Combination Index**	**0.818**	**0.696**	**0.478**	**0.408**	**0.301**
Gemcitabine(μM)	0.063	0.125	0.250	0.500	1.000
Cisplatin(μM)	0.144	0.288	0.575	1.150	2.300
CG200745(μM)	1.000	1.000	1.000	1.000	1.000

CI>1 indicates angatonism; CI=1 indicates an additive effect; and CI<1 indicates synergism. DRI>1 is favorable dose reduction that lead to toxicity reduction.

Supplementary Table 3. Combination index (CI) and dose reduction index (DRI) of CG200745 in SNU-1196/GR

a.

**SNU-1196/GR**	**Gemcitabine (μM)**		**5-FU (μM)**		
Single (IC_{50})	2.291		229.0		
**CG200745 (μM)**	0.25	0.50	0.25	0.50	1.00
Combi (IC_{50})	0.21	0.05	35.48	6.76	0.42
**Dose reduction index**	10.91	45.82	6.45	33.88	>100

b. Gemcitabine and CG200745

**Combination Index**	**0.637**	**0.589**	**0.279**	**0.083**	**0.149**	**1.140**
Gemcitabine(μM)	0.001	0.010	0.100	1.000	10.000	100.000
CG200745(μM)	0.250	0.250	0.250	0.250	0.250	0.250
**Combination Index**	**0.613**	**0.618**	**0.206**	**0.114**	**0.151**	**0.937**
Gemcitabine(μM)	0.001	0.010	0.100	1.000	10.000	100.000
CG200745(μM)	0.500	0.500	0.500	0.500	0.500	0.500

5-FU and CG200745

**Combination Index**	**0.803**	**0.620**	**0.478**	**0.356**	**0.606**	**0.785**
5-FU(μM)	0.010	0.100	1.000	10.000	100.000	1000.000
CG200745(μM)	0.250	0.250	0.250	0.250	0.250	0.250
**Combination Index**	1.062	1.037	**0.652**	**0.336**	**0.234**	**0.552**
5-FU(μM)	0.010	0.100	1.000	10.000	100.000	1000.000
CG200745(μM)	0.500	0.500	0.500	0.500	0.500	0.500
**Combination Index**	**0.856**	**0.757**	**0.522**	**0.221**	**0.177**	**0.427**
5-FU(μM)	0.010	0.100	1.000	10.000	100.000	1000.000
CG200745(μM)	1.000	1.000	1.000	1.000	1.000	1.000

CI>1 indicates angatonism; CI=1 indicates an additive effect; and CI<1 indicates synergism.
DRI>1 is favorable dose reduction that lead to toxicity reduction.

Supplementary Table 4. List of primers used in PCR analysis.

Gene	Sense	Antisense
ABCG2	TATGAGTGGCTTATCCTGCT	CACTGATCCTTCCATCTTGT
hENT1	GACAACCAGTCACCAGCCTCAG	AGAGCATCCAGCTGCACCTTCA
MRP1	CTGACAAGCTAGACCATGAATGT	TCACACCAAGCCGGCGTCTTT
MRP3	GGACCCTGCGCATGAACCTG	AGGCAAGTCCAGCATCTCTGG
MRP4	GGATCCAAGAACTGATGAGTTAAT	TCACAGTGCTGTCTCGAAAATAG
MRP5	GCTGTTCAGTGGCACTGTCAG	TCAGCCCTTGACAGCGACCTT
β–Actin	GGCATCCTCACCCTGAAGTA	GGGGTGTTGAAGGTCTCAAA

Supplementary Table 5. List of antibodies used in western blotting and immunohistochemistry

Primary antibodies	Supplier	Species	Type
Acetylated histone h3	Santa Cruz Biotechnology (Santa Cruz, CA)	Rabbit	polyclonal
C-Met	Santa Cruz Biotechnology	Rabbit	polyclonal
Caspase-3	Santa Cruz Biotechnology	Mouse	monoclonal
p21	Santa Cruz Biotechnology	Rabbit	polyclonal
BAX	Santa Cruz Biotechnology	Rabbit	polyclonal
AKT	Santa Cruz Biotechnology	Rabbit	polyclonal
ERK	Santa Cruz Biotechnology	Rabbit	polyclonal
MRP4	Santa Cruz Biotechnology	Mouse	monoclonal
HDAC7	Santa Cruz Biotechnology	Mouse	monoclonal
GAPDH	Santa Cruz Biotechnology	Mouse	monoclonal
SMAD3	Santa Cruz Biotechnology	Mouse	monoclonal
NOTCH3	Santa Cruz Biotechnology	Rabbit	polyclonal
HES5	Santa Cruz Biotechnology	Goat	polyclonal
ABCG2	Santa Cruz Biotechnology	Rabbit	polyclonal
Phospho-AKT	Cell Signaling Technology (Danvers, MA)	Rabbit	polyclonal
Phospho-MEK	Cell Signaling Technology	Rabbit	polyclonal
MEK	Cell Signaling Technology	Rabbit	polyclonal
Phospho-ERK	Cell Signaling Technology	Rabbit	polyclonal
HDAC1	Cell Signaling Technology	Mouse	monoclonal
HDAC2	Cell Signaling Technology	Mouse	monoclonal
HDAC3	Cell Signaling Technology	Mouse	monoclonal
HDAC4	Cell Signaling Technology	Rabbit	polyclonal
HDAC6	Cell Signaling Technology	Rabbit	polyclonal
YAP	Cell Signaling Technology	Rabbit	polyclonal
AXL	Cell Signaling Technology	Rabbit	polyclonal
PARP	Cell Signaling Technology	Rabbit	polyclonal
TEAD4	Abcam (Cambridge, UK)	Rabbit	polyclonal
N-cadherin	Abcam	Mouse	monoclonal
ALDH	BD Transduction Laboratories (San Jose, CA)	Mouse	monoclonal
TGF-β2	Proteintech (Rosemont, IL)	Rabbit	polyclonal
Cleaved-Caspase 3	Cell Signaling Technology	Rabbit	polyclonal
